# Maternal and/or post-weaning supplementation with *Bacillus altitudinis* spores modulates the microbial composition of colostrum, digesta and faeces in pigs

**DOI:** 10.1038/s41598-023-33175-2

**Published:** 2023-06-01

**Authors:** Ruth Rattigan, Peadar G. Lawlor, Paul Cormican, Daniel Crespo-Piazuelo, James Cullen, John P. Phelan, Samir Ranjitkar, Fiona Crispie, Gillian E. Gardiner

**Affiliations:** 1 Eco-Innovation Research Centre, Department of Science, Waterford Campus, South East Technological University, Waterford, Ireland; 2 Pig Development Department, Animal and Grassland Research and Innovation Centre, Teagasc, Moorepark, Fermoy, Co. Cork, Ireland; 3 Animal and Grassland Research and Innovation Centre, Teagasc, Grange, Dunsany, Co. Meath, Ireland; 4 APC Microbiome Ireland, University College Cork, Cork, Ireland; 5 Food Research Centre, Teagasc, Moorepark, Fermoy, Co. Cork, Ireland

**Keywords:** Antimicrobials, Applied microbiology, Bacteria, Environmental microbiology, Microbiology

## Abstract

This study examined the effects of maternal and/or post-weaning *Bacillus altitudinis* supplementation on the microbiota in sow colostrum and faeces, and offspring digesta and faeces. Sows (*n* = 12/group) were assigned to: (1) standard diet (CON), or (2) CON supplemented with probiotic *B. altitudinis* spores (PRO) from day (d)100 of gestation to weaning (d26 of lactation). At weaning, offspring were assigned to CON or PRO for 28d, resulting in: (1) CON/CON, (2) CON/PRO, (3) PRO/CON, and (4) PRO/PRO, after which all received CON. Samples were collected from sows and selected offspring (*n* = 10/group) for 16S rRNA gene sequencing. *Rothia* was more abundant in PRO sow colostrum. Sow faeces were not impacted but differences were identified in offspring faeces and digesta. Most were in the ileal digesta between PRO/CON and CON/CON on d8 post-weaning; i.e. *Bacteroidota*, *Alloprevotella*, *Prevotella*, *Prevotellaceae*, *Turicibacter*, *Catenibacterium* and *Blautia* were more abundant in PRO/CON, with *Firmicutes* and *Blautia* more abundant in PRO/PRO compared with CON/CON. *Lactobacillus* was more abundant in PRO/CON faeces on d118 post-weaning. This increased abundance of polysaccharide-fermenters (*Prevotella*, *Alloprevotella*, *Prevotellaceae*), butyrate-producers (*Blautia*) and *Lactobacillus* likely contributed to previously reported improvements in growth performance. Overall, maternal, rather than post-weaning, probiotic supplementation had the greatest impact on intestinal microbiota.

## Introduction

In commercial pig production, weaning is a challenging period associated with negative impacts on pig growth and health^1^. During this period, piglets are exposed to psychological, environmental and nutritional stressors^2,3^, which often result in a heightened susceptibility to post-weaning diarrhoea (PWD) and reduced growth performance^2^. Antibiotics and zinc oxide (ZnO) are frequently added to the diet of weaned pigs to prevent these issues. However, the use of antibiotics for growth promotion was banned in the EU in 2006 due to the rise in antimicrobial resistance (EC Regulation no. 1831/2003). Further restrictions, including a ban on the preventative use of antibiotics in groups of animals and via medicated feed, and a ban on the use of pharmacological doses of ZnO, came into effect in the EU in January and June 2022, respectively (Regulation [EU] 2019/6 on Veterinary Medicines and Regulation [EU] 2019/4 on Medicated Feed). As a result, there is an urgent need to develop suitable alternative dietary strategies to maintain pig productivity and well-being during the weaning transition, with probiotics being one promising approach.

Probiotics are defined as ‘live microorganisms which, when administered in adequate amounts, confer a health benefit on the host’^4^. *Bacillus* spp. are spore formers and as a result offer some advantages over other probiotic micro-organisms commonly used in livestock i.e. *Lactobacillus*, *Enterococcus* and *Saccharomyces*. The ability to form endospores affords *Bacillus* spp. greater resistance to the harsh conditions encountered during product formulation and feed processing activities, such as spray drying and pelleting (e.g. low moisture and high temperatures), thereby enhancing viability and shelf-life^5^. Furthermore, studies have shown that *Bacillus* spores can resist the acidic conditions in the stomach, thereby facilitating transit through the gastrointestinal tract (GIT)^6,7^.

Studies such as those conducted by Dou et al.^8^, which found that the faecal microbiota of pigs that developed PWD differed from that of healthy pigs from as early as 7 days of age, demonstrate the importance of early-life microbiota. This has led to the current thinking that manipulation of the microbiota early in life should have the greatest impact^9^. Maternal supplementation with feed additives is one strategy that can potentially result in beneficial effects in offspring earlier in life than can be achieved with direct supplementation. Using this maternal supplementation strategy, *Bacillus*-based probiotics have demonstrated a range of benefits including reduced enteric pathogens in suckling pigs^10,11^, enhanced piglet growth rates^10–12^ and reduced incidence of diarrhoea^12^. Recently, our research group has demonstrated lifetime growth benefits in the offspring of sows supplemented with *Bacillus altitudinis* WIT588 spores during late gestation and lactation^13^. Increased body weight was observed in the offspring of *Bacillus-*supplemented sows during the finishing period, ultimately leading to increased carcass weight and kill-out percentage at slaughter^13^. Proposed mechanisms of action include improved colostrum quality in sows and enhanced small intestinal absorptive capacity in offspring during the critical early post-weaning (PW) period, leading to an observed improvement in feed conversion efficiency^13^. Modulation of the intestinal microbiota may also be a factor, considering its contribution to intestinal health and nutrient utilization^14^. Thus, the objective of this study was to determine, for the first time, whether maternal and/or PW supplementation with *B. altitudinis* WIT588 spores influences the composition and/or diversity of the microbiota in sow faeces and colostrum and in the offspring digesta and faeces, at various time-points from late gestation through to the end of the finishing period.

## Results

For simplification purposes, sow treatment groups are abbreviated throughout the remainder of the results section as follows: CON, sows fed a control diet; and PRO, sows fed a probiotic-supplemented diet. Similarly, offspring treatment groups are abbreviated as follows: CON/CON, piglets weaned from control sows, fed a control diet; CON/PRO, piglets weaned from control sows, fed a probiotic-supplemented diet; PRO/CON, piglets weaned from probiotic-supplemented sows, fed a control diet; and PRO/PRO, piglets weaned from probiotic-supplemented sows, fed a probiotic-supplemented diet.

### Effects on colostral microbiota

#### Colostral microbial diversity

**Figure 1:**
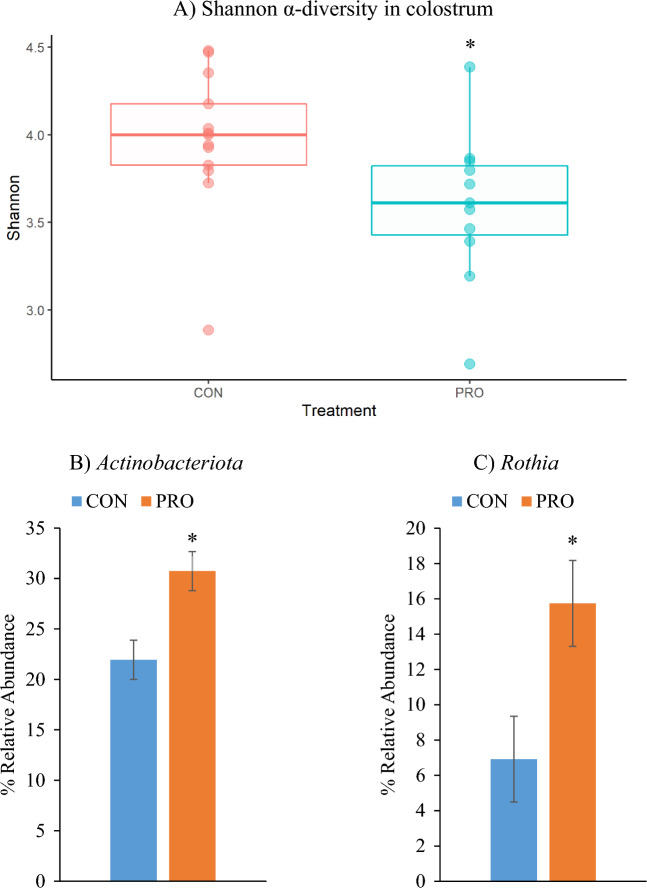
The effect of sow treatment on Shannon α-diversity (**A**), and the mean relative abundance of *Actinobacteriota* (**B**) and *Rothia* (**C**) within the sow colostrum. Maternal treatment: *CON* control and *PRO* probiotic. Significant differences between treatments within each sampling time point are indicated as *P < 0.05.

The effect of treatment on the colostral microbial diversity is presented in Fig. 1a. Shannon α-diversity, which is a measure of species richness and evenness, was lower in the colostrum of PRO sows compared with the CON sows (*P* < 0.05). In relation to β-diversity in the colostrum, there was no clustering based on treatment (Supplementary Fig. S1).

#### Colostral microbiota composition

A total of 15 phyla, 82 families and 190 genera were identified in the colostrum samples. *Firmicutes* and *Actinobacteriota* were the predominant phyla (Supplementary Fig. S2a), while the predominant genera were *Staphylococcus*, *Corynebacterium* and *Rothia* (Supplementary Fig. S2b). Differentially abundant taxa are presented in Supplementary Table S1 and the mean relative abundance of bacterial phyla and genera are presented in Supplementary Fig. S2a and S2b, respectively. At phylum level, *Actinobacteriota* was more abundant in the colostrum of the PRO sows compared with the CON sows (*P* < 0.05; Fig. 1b). At genus level, colostrum from the PRO sows had a greater abundance of *Rothia* (*P* < 0.05; Fig. 1c) and *Globicatella* (*P* < 0.01), and a lower abundance of *Vagococcus* (*P* < 0.01) compared with the CON sows. A BLAST search of the sequence assigned to the genus *Rothia* resulted in several matches including *R. marina, R. endophytica, R. nasimurium* and *R. mucilaginosa*.

### Effects on sow faecal microbiota

#### Sow faecal microbial diversity

There was no effect of treatment on the Shannon α-diversity at any sampling point in the sow faecal samples (Supplementary Fig. S3a). In general, microbial diversity appeared to decline during the lactation period, regardless of sow treatment. The β-diversity of the faecal microbiota is represented using an unweighted UniFrac multidimensional scaling (MDS) plot (Supplementary Fig. S3b). While samples do not cluster according to treatment (control or probiotic), they do separate based on sampling time-point, with samples collected during gestation and at farrowing clustering separately to those collected during lactation (day (d) 13 of lactation and weaning).

#### Sow faecal microbiota composition

A total of 15 phyla, 73 families and 157 genera were identified in the sow faeces across all sampling points. *Firmicutes* and *Bacteroidota* were the predominant phyla found at all sampling points (Supplementary Fig. S4) and *Spirochaetaceae*, *Prevotellaceae*, *Oscillospiraceae* and *Rikenellaceae* were the predominant families (data not shown). There were no differences due to probiotic supplementation found at phylum, family or genus level in the sow faecal samples at any sampling point.

### Effects on offspring faecal and digesta microbiota

#### Offspring faecal and digesta microbial diversity

**Figure 2:**
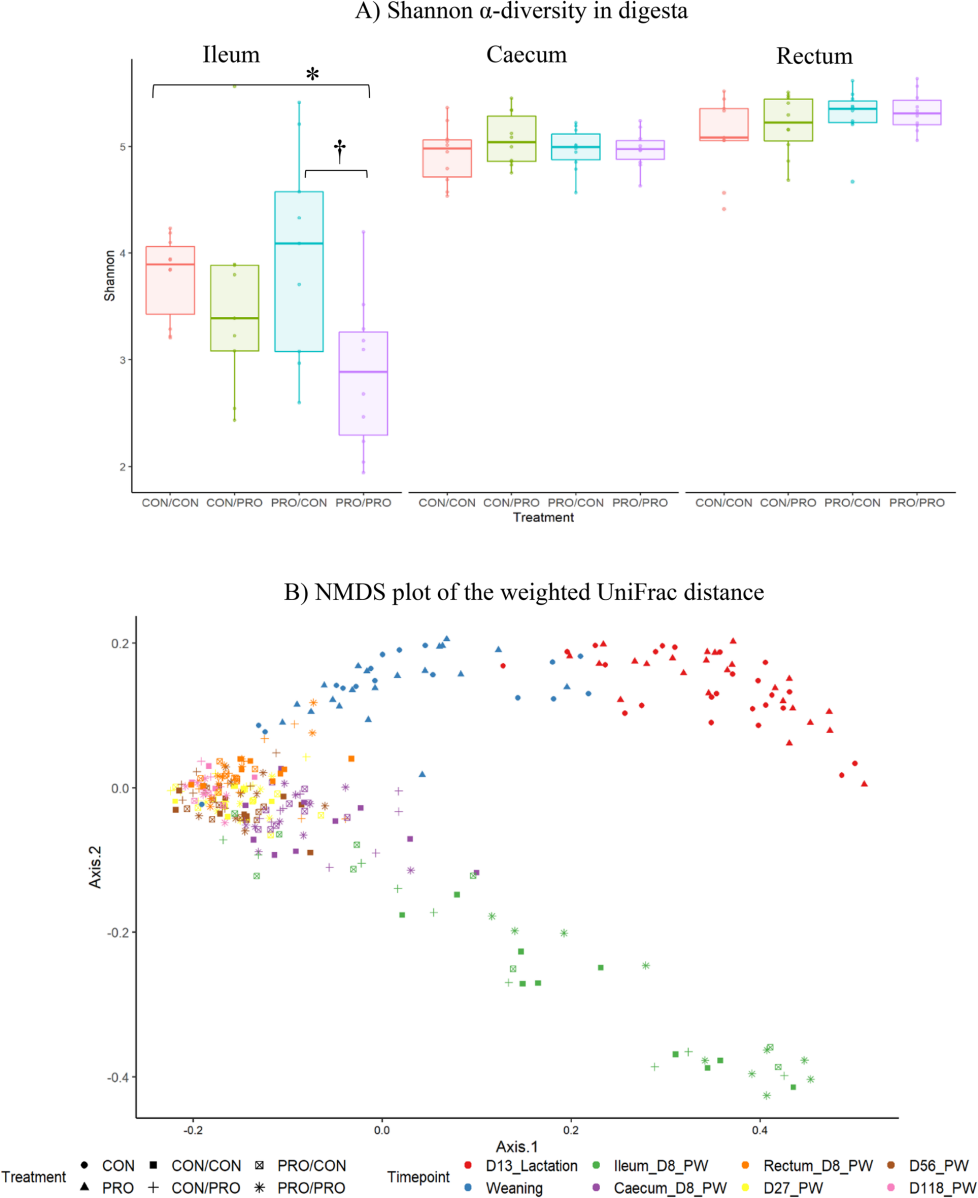
Effect of maternal and post-weaning treatment on Shannon α-diversity in offspring digesta (**A**) and on the weighted UniFrac distances (β-diversity) of the microbiota in offspring faeces and digesta at all sampling points (**B**). Treatments are as follows (maternal treatment/post-weaning treatment): CON/CON, CON/PRO, PRO/CON and PRO/PRO; where *CON* is control and *PRO* is probiotic. Significant differences between treatments within each sampling time point are indicated as *P < 0.05 and ^†^0.05 < *P* < 0.10. Colours indicate the sample type and time-point at which pigs were sampled (samples collected at the weaning, d27_PW, d56_PW and d118_PW time points are faecal samples). NMDS, non-metric multidimensional scaling.

There was no difference in α-diversity in offspring faecal samples across sampling time-points (*P* > 0.05; Supplementary Fig. S5). There was an effect of treatment on microbial α-diversity in the ileum (Fig. 2a), with Shannon diversity reduced in the PRO/PRO group compared with the CON/CON group (*P* < 0.05) and PRO/CON groups (*P* = 0.05), although the latter was a tendency. There were no treatment-related differences in microbial diversity in the caecum or rectum.

The β-diversity of the faecal and digesta microbiota is represented using a weighted UniFrac non-metric MDS (nMDS) plot to assess the differences between treatment groups (Fig. 2b). Overall, there were several clusters depending on sampling point/sample type. Interestingly, faecal samples collected on d13 of lactation clustered separately to faecal samples collected on the day of weaning (d26 of lactation). These two groups also clustered separately from samples collected PW. Faecal/digesta samples collected on d8, d27, d56 and d118 PW do not seem to segregate, with the exception of ileal digesta samples from d8 PW, which cluster separately to the other samples/sampling time-points. Permutational ANOVA showed that the weighted UniFrac metric for the ileal digesta on d8 PW differed to the weighted UniFrac metric for the other faecal/digesta samples (*P* < 0.05). Similarly, the weighted UniFrac metric tended to differ between the faeces at weaning and the other faecal/digesta samples (*P* = 0.08) and between the caecum on d8 PW and the other faecal/digesta samples (*P* = 0.08).

#### Offspring faecal and digesta microbiota composition

**Figure 3:**
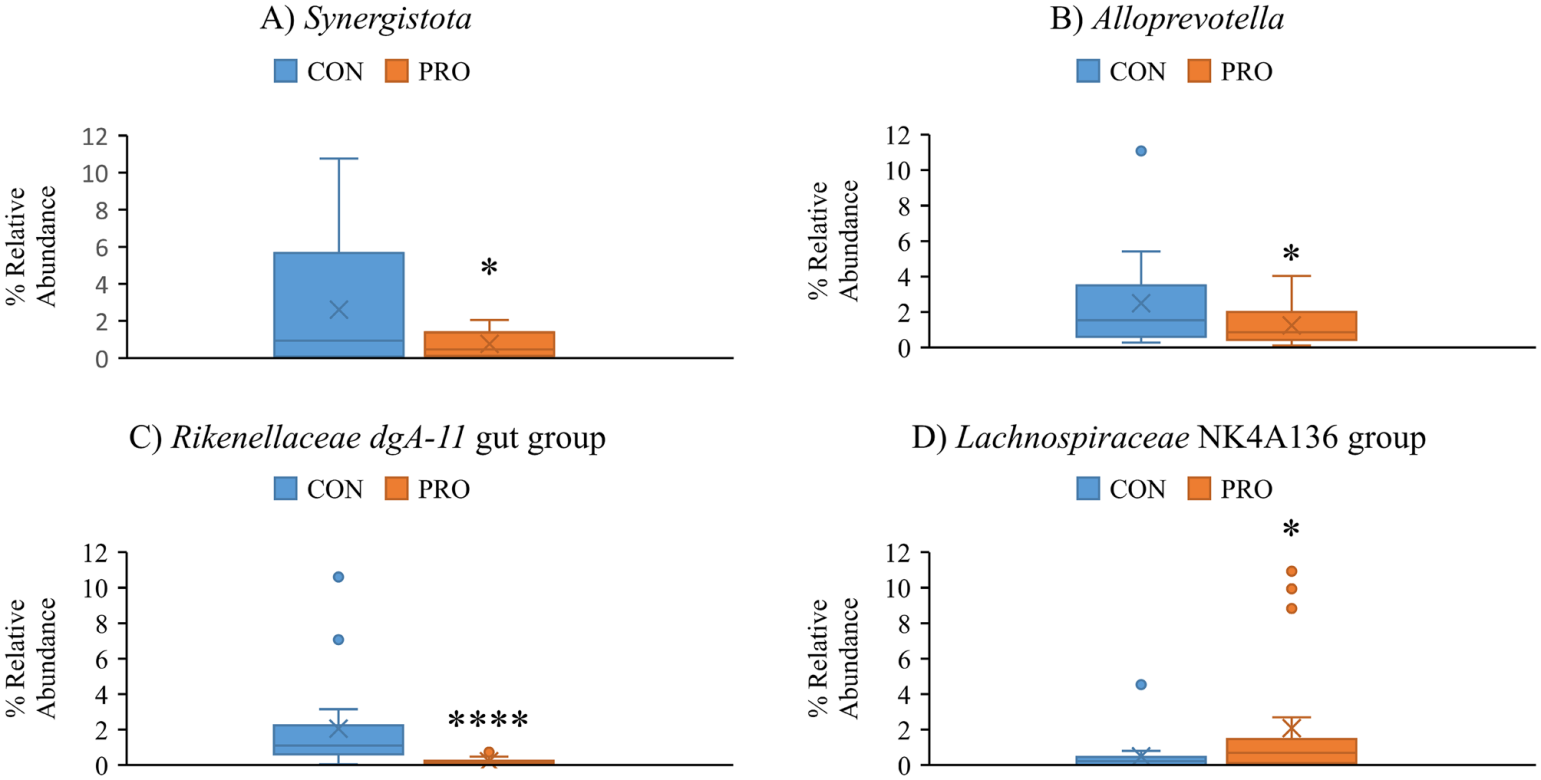
The effect of maternal treatment on the mean relative abundance (%) of *Synergistota* (**A**), *Alloprevotella* (**B**), *Rikenellaceae dgA-11 gut group* (**C**) and *Lachnospiraceae NK4A136 group* (**D**) in the faeces of offspring at weaning (day 26 lactation). Maternal treatment: *CON* control, *PRO* probiotic. Significant differences between treatments are indicated as *P < 0.05; **P < 0.01; ***P < 0.001; ****P < 0.0001.

**Figure 4:**
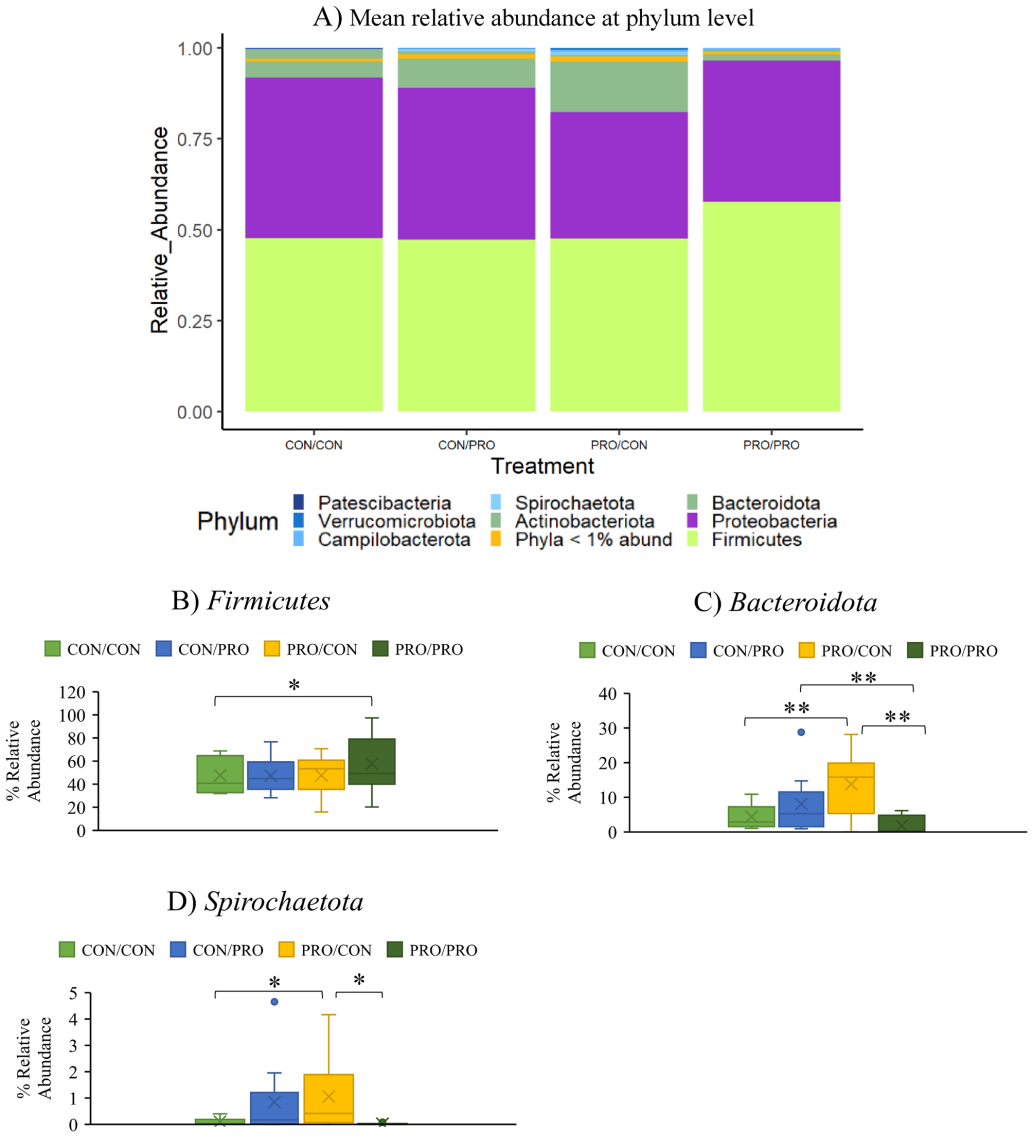
The effect of maternal and post-weaning treatment on the mean relative abundance (%) of bacterial phyla (**A**), *Firmicutes* (**B**), *Bacteroidota* (**C**) and *Spirochaetota* (**D**) in the ileum of offspring on day 8 post-weaning. Treatments are as follows (maternal treatment/post-weaning treatment): CON/CON, CON/PRO, PRO/CON and PRO/PRO; where *CON* is control and *PRO* is probiotic. Significant differences between treatments are indicated as *P < 0.05, **P < 0.01.

**Figure 5:**
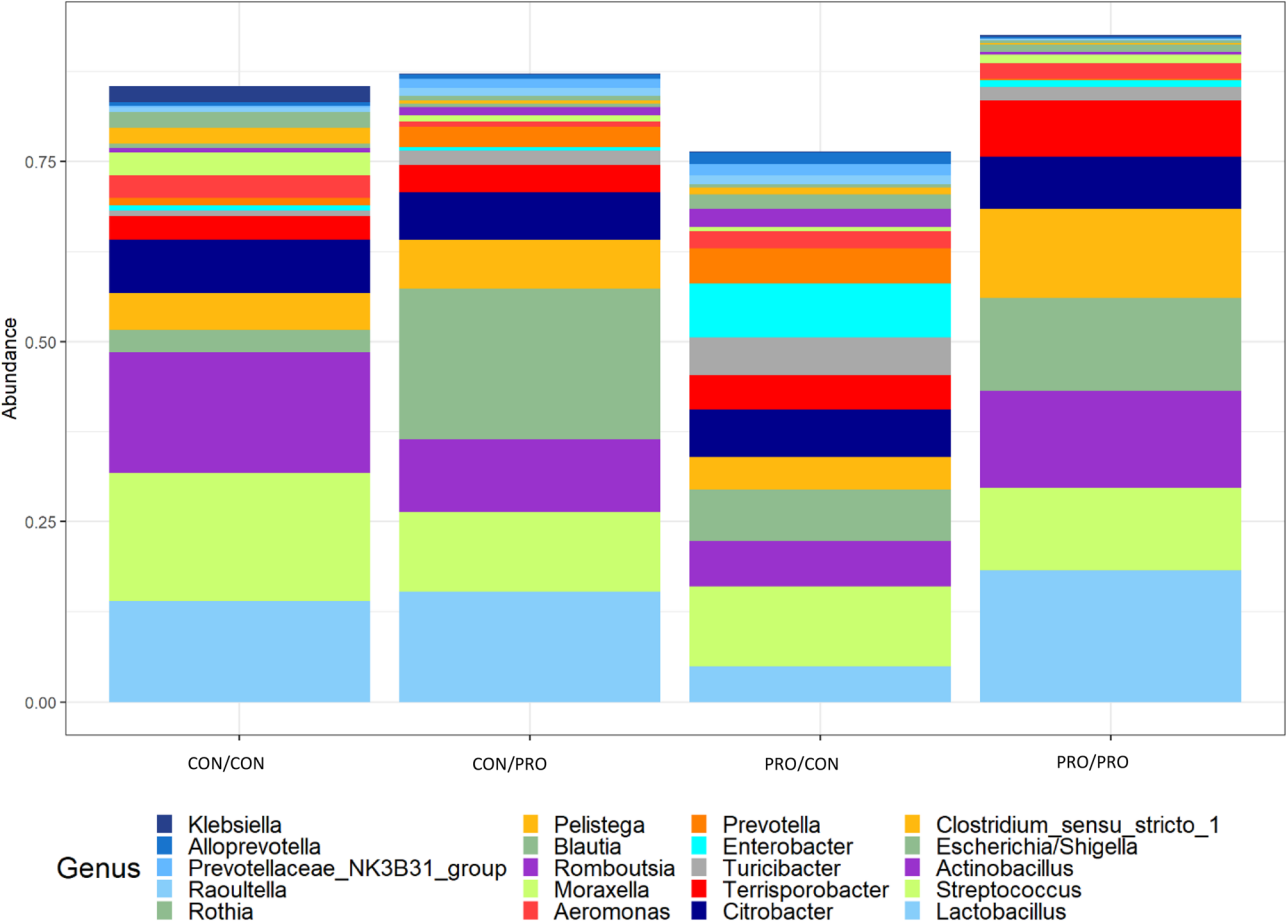
The effect of maternal and post-weaning treatment on the mean relative abundance of the 20 most abundant genera in the ileum of offspring on day 8 post-weaning. Treatments are as follows (maternal treatment/post-weaning treatment): CON/CON, CON/PRO, PRO/CON and PRO/PRO; where *CON* is control and *PRO* is probiotic.

**Figure 6:**
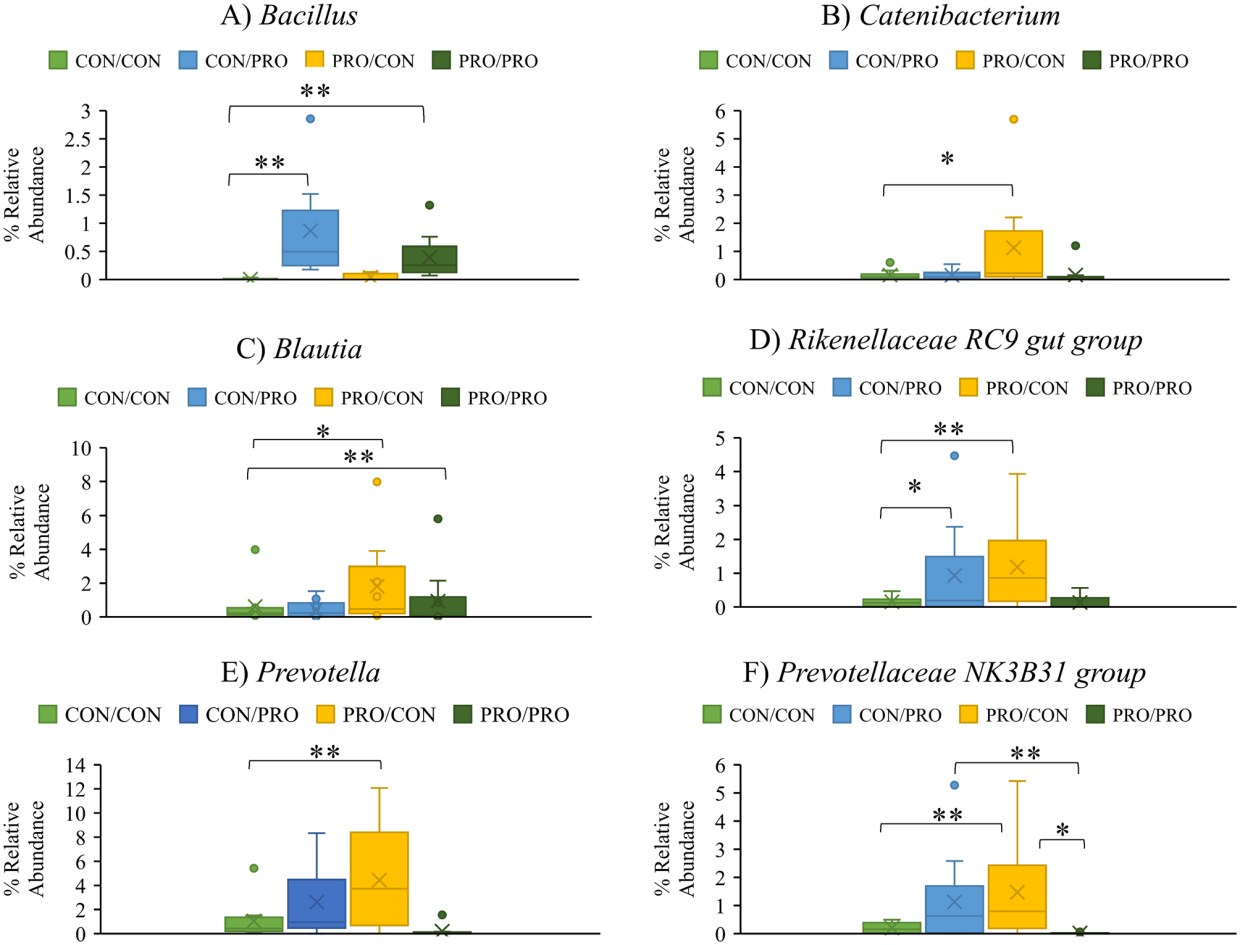
The effect of maternal and post-weaning treatment on the mean relative abundances of *Bacillus* (**A**), *Catenibacterium* (**B**), *Blautia* (**C**)*, Rikenellaceae RC9 gut *group (**D**), *Prevotella* (**E**) and *Prevotellaceae NK3B3A* group (**F**) in the ileum on day 8 post-weaning. Treatments are as follows (maternal treatment/post-weaning treatment): CON/CON, CON/PRO, PRO/CON and PRO/PRO; where *CON* is control and *PRO* is probiotic. Significant differences between treatments are indicated as *P < 0.05, **P < 0.01*.*

**Figure 7:**
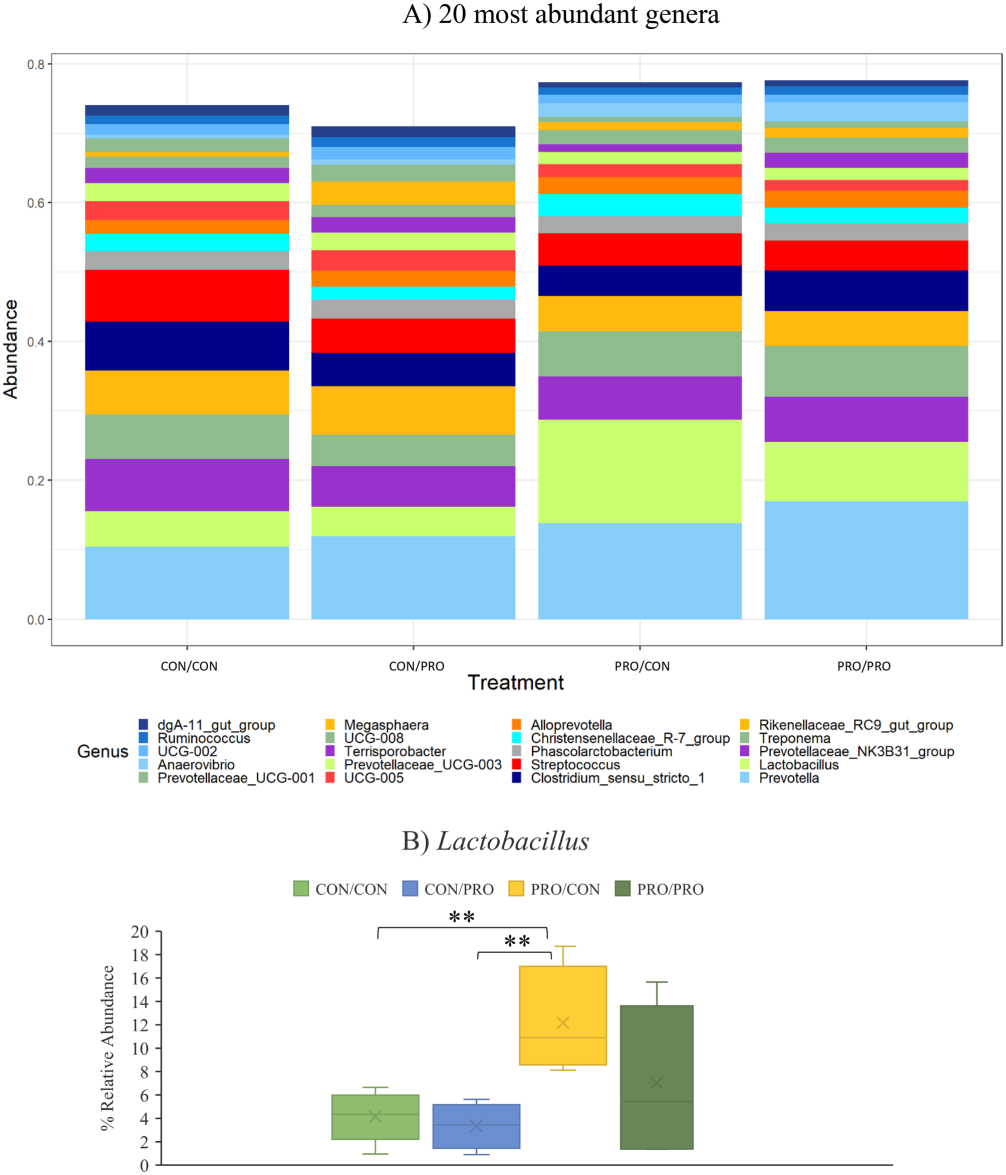
The effect of maternal and post-weaning treatment on the mean relative abundances (%) of the 20 most abundant genera (**A**) and *Lactobacillus* spp. (**B**) in the faeces of offspring on day 118 post-weaning. Treatments are as follows (maternal treatment/post-weaning treatment): CON/CON, CON/PRO, PRO/CON, and PRO/PRO; where *CON* is control and *PRO* is probiotic. Significant differences between treatments are indicated as *P < 0.05, **P < 0.01.

A total of 16 phyla, 99 families and 248 genera were identified in offspring faecal and digesta samples across all sampling points, with *Firmicutes* and *Bacteroidota* the predominant phyla. Up to and including d8 PW, the third most abundant phylum was *Proteobacteria*, which, as expected, increased in the early PW period (d8 PW) and declined thereafter. All differences identified between treatment groups pre-weaning [lactation d13 and d26 (weaning)] are presented in Supplementary Table S2 and all treatment-related differences in samples collected PW are presented in Supplementary Table S3. The differentially abundant phyla and genera in the faeces of offspring at weaning are presented in Fig. 3. The mean relative abundance (%) of all bacterial phyla and selected differentially abundant phyla in the offspring ileal digesta on d8 PW are presented in Fig. 4. The 20 most abundant genera, and selected differentially abundant genera in the ileum on d8 PW are presented in Figs. 5 and 6, respectively. The 20 most abundant genera in offspring faeces and the mean relative abundance of *Lactobacillus* on d118 PW are presented in Fig. 7.

#### Pre-weaning

There were no differences in the abundance of taxa above 1% relative abundance pre-weaning. At weaning (d26 of lactation), the *Synergistota* phylum was less abundant in the faeces of pigs weaned from the PRO sows compared with pigs weaned from the CON sows (*P* < 0.05; Fig. 3a). At genus level, *Alloprevotella* (*P* < 0.05; Fig. 3b) and *Rikenellaceae* dgA-11 gut group (*P* < 0.001; Fig. 3c) were less abundant, and *Lachnospiraceae NK4A136* (*P* < 0.05; Fig. 3d) was more abundant in pigs weaned from the PRO sows compared with pigs weaned from the CON sows.

#### Post-weaning

##### Ileum d8 PW

The mean relative abundance of all bacterial phyla in the ileum is presented in Fig. 4a. There were six differentially abundant phyla identified (Supplementary Table S3). *Actinobacteriota* was less abundant in the CON/PRO and PRO/CON groups compared with the CON/CON group (*P* < 0.01; Fig. 4a). *Firmicutes* was more abundant in the PRO/PRO group compared with the CON/CON group (*P* < 0.05; Fig. 4a,b). *Bacteroidota* was more abundant in the PRO/CON group compared with the CON/CON and PRO/PRO groups (*P* < 0.01), and more abundant in the CON/PRO group compared with the PRO/PRO group (*P* < 0.01; Fig. 4a,c). *Spirochaetota* was more abundant in the PRO/CON group compared with the CON/CON group and PRO/PRO groups (*P* < 0.05 and *P* < 0.01, respectively; Fig. 4a,d).

Fifty-three treatment-related differences were identified at genus level (Supplementary Table S3). For simplification purposes, only significant differences for genera with a relative abundance > 1% are reported here with the exception of the *Bacillus* genus. The mean relative abundance of the 20 most abundant genera is presented in Fig. 5. *Chryseobacterium* was less abundant in the CON/PRO, PRO/CON and PRO/PRO groups compared with the CON/CON group (*P* < 0.01, *P* < 0.05 and *P* < 0.001, respectively; Supplementary Table S3). *Alloprevotella* was more abundant in the PRO/CON group compared with the CON/CON and PRO/PRO groups (*P* = 0.05 and *P* < 0.05, respectively; Fig. 5). *Turicibacter* was more abundant in the PRO/CON group compared with the CON/CON group (*P* < 0.01; Fig. 5). *Pelistega* was less abundant in the CON/PRO group compared with the CON/CON group (*P* < 0.05; Fig. 5). *Rothia* was less abundant in the PRO/CON and PRO/PRO groups compared with the CON/CON group (*P* < 0.05; Fig. 5). *Terrisporobacter* and *Clostridium sensu stricto 1* were more abundant in the PRO/PRO group compared with the CON/CON group (*P* < 0.05; Fig. 5).

*Escherichia/Shigella* was more abundant in the CON/PRO, PRO/CON and PRO/PRO groups compared with the CON/CON group (*P* < 0.01; Fig. 5). A BLAST search of the sequence assigned to the genus *Escherichia/Shigella* resulted in several matches including numerous *E. coli* strains*, S. sonnei, S. flexneri, E. fergusonii* and *Escherichia* spp*.*

*Bacillus* was more abundant in the CON/PRO and PRO/PRO groups compared with the CON/CON group (*P* < 0.001 and *P* < 0.01, respectively; Fig. 6a). *Catenibacterium* was more abundant in the PRO/CON group compared with the CON/CON group (*P* < 0.05; Fig. 6b). *Blautia* was more abundant in the PRO/CON and PRO/PRO groups compared with the CON/CON group (*P* < 0.05 and *P* < 0.01, respectively; Fig. 6c). *Rikenellaceae RC9* gut group was more abundant in the CON/PRO and PRO/CON groups compared with the CON/CON group (*P* < 0.05; Fig. 6d). *Prevotella* (*P* < 0.01; Fig. 6e) was more abundant in the PRO/CON group compared with the CON/CON group. *Prevotellaceae NK3B31* was more abundant in the PRO/CON group compared with the CON/CON and PRO/PRO groups (*P* < 0.01 and *P* < 0.05) and more abundant in the CON/PRO group compared with the PRO/PRO group (*P* < 0.01; Fig. 6f).

##### Caecum d8 PW

Three differentially abundant genera were identified in the caecum on d8 PW (Supplementary Table S3). *Treponema* was more abundant in the PRO/CON group compared with the CON/CON group (*P* < 0.05).

##### Rectum d8 PW

Two differentially abundant phyla were identified in the rectum on d8 PW (**S**upplementary Table S3). *Campylobacterota* was more abundant in the CON/PRO group compared with the CON/CON, PRO/CON and PRO/PRO groups (*P* < 0.01).

##### Faeces d27 PW

Three differences at phylum level and two differences at genus level were identified in the faecal samples collected on d27 PW (Supplementary Table S3). The *Firmicutes* phylum was less abundant in the CON/PRO group compared with the PRO/CON and PRO/PRO groups (*P* < 0.05). The *Prevotellaceae UCG 001* genus was more abundant in the PRO/CON and PRO/PRO groups compared with the CON/CON group (*P* < 0.01). The *Lachnospiraceae ND3007* group genus was less abundant in the PRO/PRO group compared with the CON/CON and CON/PRO groups (*P* < 0.001 and *P* < 0.05, respectively).

##### Faeces d56 PW

There was one differentially abundant genus in the faecal samples collected on d56 PW (Supplementary Table S3); *Alloprevotella* was less abundant in the PRO/PRO group compared with the PRO/CON group (*P* < 0.05).

##### Faeces d118 PW

Eight differentially abundant genera were identified in the faecal samples collected on d118 PW (Supplementary Table S3). *Lactobacillus* (Fig. 7a,b) was more abundant in the PRO/CON group compared with the CON/CON and CON/PRO groups (*P* < 0.01). *Succinivibrio* and *Lachnospiraceae NK4A136* group were less abundant in the PRO/CON group compared with the CON/PRO group (*P* < 0.05).

### Effects on short chain fatty acid (SCFA) concentrations in the ileum on d8 PW

The effect of treatment on the concentration of SCFA in the ileum is presented in Table 1. There was no effect of treatment on the concentration of total SCFA, total volatile fatty acids (VFA), total branched chain fatty acids (BCFA), lactic acid or propionic acid in the ileal digesta. However, the concentration of acetic acid was higher (5.62 vs 2.93 mmol/kg, SEM 0.912, *P* < 0.05) and the concentration of valeric acid tended to be lower (0.021 vs 0.042 mmol/kg, SEM 0.006, *P* = 0.099) in the ileal digesta of pigs receiving the probiotic PW compared with the un-supplemented pigs. There was also a tendency for a maternal treatment effect on the concentration of butyric acid, with pigs weaned from the probiotic-supplemented sows having a higher concentration compared with those weaned from un-supplemented sows (0.174 vs 0.100 mmol/kg, SEM 0.030, *P* = 0.07).

**Table 1:** Effect of maternal and post-weaning treatment on the concentrations of short chain fatty acids (mmol/kg digesta) in the offspring ileal digesta on day 8 post-weaning.

	Treatment				SEM	*P* values		
	CON/CON	CON/PRO	PRO/CON	PRO/PRO		Maternal	Post-weaning	Maternal × Post-weaning
SCFA	12.35	15.04	12.63	15.30	2.571	0.915	0.302	0.988
VFA	4.27	4.22	3.85	5.68	1.553	0.791	0.602	0.582
BCFA	0.121	0.104	0.128	0.119	0.0028	0.646	0.574	0.840
Lactic acid	8.08	8.91	8.79	9.63	1.382	0.611	0.550	0.984
Acetic acid	3.41	5.96	2.52	5.32	1.295	0.499	0.041	0.761
Propionic acid	0.029	0.012	0.000	0.042	0.0396	0.984	0.983	0.980
Butyric acid	0.097	0.103	0.124	0.244	0.0446	0.085	0.241	0.335
Valeric acid	0.040	0.044	0.045	0.01	0.0109	0.108	0.099	0.069

*SCFA* short chain fatty acid, *VFA* volatile fatty acids, *BCFA* branched chain fatty acids; Differences between treatments are significant for *P* ≤ 0.05, while 0.05 < *P* < 0.10 is a tendency towards significance.

Treatments are as follows (maternal treatment/post-weaning treatment): CON/CON, CON/PRO, PRO/CON and PRO/PRO; where *CON* is control and *PRO* is probiotic.

Statistical analysis was performed using the GLIMMIX procedure of SAS 9.4 and the ilink function was used to back-transform the data to the original scale. The model included the effects of maternal and post-weaning treatment as fixed effects and their associated interaction. The individual pig was the experimental unit. The least square means were compared between the treatment groups using the *t* test and the *P*-values were corrected for multiple comparisons using the Tukey adjustment.

## Discussion

To the authors’ knowledge, this is the first study examining the effects of maternal supplementation with a *Bacillus*-based probiotic on the microbial composition of the sow’s colostrum and faeces and offspring digesta and faeces from suckling through to the finishing period. The results show that maternal probiotic supplementation influences the microbial composition of the sows’ colostrum but not the sows’ faeces. Supplementation, either maternally or directly PW, also influenced the microbial composition of offspring digesta and faeces PW. However, most of the effects observed were in pigs weaned from probiotic-supplemented sows, with a number of potentially beneficial effects observed in the ileal digesta of pigs in the PRO/CON group on d8 PW and in the faeces of this group on d118 PW. *Bacillus* spp. were detected in the ileal digesta of both maternally and directly treated piglets on d8 PW, albeit at < 1% abundance. The abundance of *Bacillus* was higher in the ileum of pigs receiving the probiotic at the time of sampling (0.87% and 0.39% in the CON/PRO and PRO/PRO groups, respectively vs 0.01%, in the PRO/CON group). This finding is in agreement with counts of the administered strain, whereby the probiotic was detected in the ileal digesta of the CON/PRO and PRO/PRO groups but not the PRO/CON group on d8 PW, as reported previously in our parallel publication^13^. Similarly, when a combination of *Bacillus subtilis* and *B. licheniformis* was administered to growing pigs, Kaewtapee et al.^15^ noted that operational taxonomic units (OTUs) corresponding to the *Bacillus* genus appeared at < 1% abundance in the ileum of both supplemented and un-supplemented pigs; however, they did not find the difference in abundance between treatment and control animals as we do here. Those authors suggested that the detection of *Bacillus* within the ileum indicates that they are able to germinate in the mammalian GIT, albeit at a low rate and thus may be unable to persist^15,16^. Together, these findings suggest that *Bacillus* probiotics do not predominate within the small intestinal microbiota, but that administered spores do appear to germinate, as they are detected using DNA-based methods, suggesting the presence of vegetative cells, which are more amenable to DNA extraction than spores^17^.

As described previously, *B. altitudinis* WIT588 was detected in the faeces of supplemented compared to unsupplemented sows and the presence of the probiotic in the faeces is the likely mode of transmission of the probiotic between the sow and her offspring^13^. However, the 16S rRNA gene sequencing analysis conducted here did not find any differences in the sows’ faecal bacterial diversity or composition. Interestingly, the sow faecal microbiome during gestation and at farrowing differed from that observed during lactation. This is in agreement with the findings of previous studies^18–20^ and is likely due to dietary, metabolic and hormonal changes during lactation.

Although sow faecal bacterial diversity and composition were not impacted by probiotic supplementation, both parameters were altered in the colostrum of probiotic-supplemented sows. This may be due to the lower bacterial diversity in colostrum compared with faeces, as greater community diversity is associated with increased resistance to alterations in composition^21,22^. Probiotic supplementation led to a reduction in bacterial diversity in the colostrum. Whilst, a reduction in diversity within the gut microbiome is considered undesirable, little is known about the effects of reduced microbial diversity in colostrum, which intrinsically has a smaller microbial community with fewer functions. Hence, a high level of microbial diversity may not be necessary within the colostrum microbiome. Furthermore, loss of diversity does not always equate to a loss in microbial function. Soil microbial communities, for example, may recover function even if microbial diversity remains reduced^21^. Importantly, the reduction in bacterial diversity in colostrum observed in the present study due to probiotic administration was not associated with any negative effects on offspring growth or health in the pre- or post-weaning periods as reported in a parallel study of growth performance already published^13^.

Although few studies are available for comparison, the predominant phyla (*Firmicutes* and *Actinobacteria*) and genera (*Staphylococcus*, *Corynebacterium* and *Rothia*) identified in the colostrum in this study are in line with previous observations for sow colostrum and milk^23^. The higher relative abundance of the phylum *Actinobacteria* in the colostrum of probiotic-supplemented compared to control sows, likely resulted from the increased abundance of the *Rothia* genus. *Rothia* spp. are common oral, skin, and intestinal commensals of humans, pigs, and rodents^24–26^ and have previously been found in the milk of pigs, humans, goats and rats^24,25^. As *Rothia* spp. are found in the oral cavity and on the skin, it is possible they originated from the sow’s teats or the piglets’ mouths^27,28^; however, teats were cleaned prior to sampling in order to prevent contamination from the skin. The sequence identified as differentially abundant in the colostrum matches with several *Rothia* spp*.,* classified as environmental (*R. marina and endophytica*), but also animal species (*R. nasimurium*) and one species which is considered an opportunistic pathogen in humans (*R. mucilaginosa*). The observed differences in microbial composition in the colostrum may be due to the altered nutrient composition of the colostrum from probiotic-supplemented sows, which had a higher concentration of protein and a lower concentration of lactose than colostrum from non-supplemented sows as reported previously in our parallel publication^13^. Alterations in colostrum composition may be caused by improvements in nutrient digestion and absorption, associated with improved intestinal health and/or enzyme activity in the sow GIT resulting from probiotic supplementation^29^. *Rothia* spp. can ferment carbohydrates and proteins^30,31^ and a study found that growth of the oral commensal *Rothia mucilaginosa* was reduced when culture medium was supplemented with lactose compared with glucose or sucrose^30^. Thus, the lower abundance of *Rothia* in the colostrum from the control sows may be associated with the higher concentration of lactose in their colostrum. Another possible explanation for the altered microbial composition in the colostrum of probiotic-supplemented sows is bacterial translocation from the sow’s GIT to the mammary gland via the bloodstream. This is referred to as the enteromammary pathway. A number of studies in pigs, cows, humans and mice have provided evidence to support this theory^32–34^. However, in order to see changes in the colostrum composition via this route, the sow intestinal microbiota would first need to be impacted and we cannot ascertain this, as it was not possible to take intestinal samples from the sows i.e. all that we can conclude is that the faecal microbiota was not impacted. Nonetheless, it is likely that the intestinal microbiota of the sows has been modulated by the probiotic, as it was within the ileum microbiota that most impact was seen in the offspring.

It was expected that maternal probiotic supplementation would influence the composition of the offspring microbiota whilst they were still suckling the sow and in the early PW period. However, most effects were detected in the ileal digesta on d8 PW and in faecal samples collected PW and in the finisher period. There are very few studies in which *Bacillus*-based probiotics have been supplemented to sows and of these, even fewer have measured the effects on the microbiota of the offspring. Baker et al.^10^ found beneficial alterations to the ileal and colonic microbiota of offspring on d3 and d10 of lactation following maternal *Bacillus* supplementation, using the now outdated terminal restriction fragment length polymorphism technique. Considering the large number of differences in bacterial abundance identified in the ileal digesta on d8 PW in the present study, it is possible that differences may also have been detected there during the suckling period. However, intestinal contents were not collected from offspring during lactation.

The changes observed within the gut microbiota of offspring from probiotic-supplemented sows during the early PW period may be related to the altered colostrum microbiome, as it has been shown that the early-life intestinal microbiota of pigs is strongly influenced by the maternal milk microbiota^35^. At weaning, pigs weaned from the probiotic-supplemented sows had a higher abundance of *Lachnospiraceae,* a family associated with fermentation of plant polysaccharides and the production of butyric acid^36^. Butyric acid is the preferential energy source of the colonocytes^37^. It may enhance intestinal health by facilitating the absorption of water, sodium and potassium, regulating gene expression by inhibiting the expression of histone deacetylases, enhancing the expression of host defence peptides, and via its antibacterial activity^38,39^. Therefore, the increased abundance of *Lachnospiraceae* in the offspring from probiotic-supplemented sows may have benefits in terms of intestinal integrity and nutrient absorption. This may help to explain the previously reported positive modulation of small intestinal histology parameters at d8 PW, improved feed efficiency observed during the first 14 days PW and the subsequent improvements in growth in the offspring from probiotic-supplemented sows^13^. Although VFA were not measured at weaning, there was a tendency for an increase in butyric acid in the ileal digesta collected on d8 PW in pigs weaned from the probiotic-supplemented sows. This may have contributed to the previously reported improved villous height in the duodenum and ileum of offspring weaned from the probiotic-supplemented sows observed at d8 PW^13^. While butyric acid is the preferential energy source of the colonocytes, it can also stimulate cell proliferation and growth in the small intestine (as reviewed by Liu^40^). Oral supplementation with various forms of butyrate to weaned pigs resulted in improved small intestinal morphology^41–45^, increased digestive enzyme activity^44^ and reduced incidence of diarrhoea^43^. Acetic acid was elevated in the ileal digesta of piglets receiving the probiotic PW. Acetic acid can be metabolized by peripheral tissues and provide energy for the host^46^. Thus, this increased ileal acetic acid concentration could contribute to improved energy salvage from the diet, albeit no growth improvements were seen in these animals in our parallel study^13^.

Most probiotic-mediated effects were found within the ileal microbiota of offspring at d8 PW, and most differences occurred between the pigs weaned from probiotic-supplemented sows and offered the unsupplemented diet PW (PRO/CON) and pigs weaned from the unsupplemented sows and offered the unsupplemented diet PW (CON/CON) groups. At this time, the microbiota may be more plastic as a result of disruptions caused by the change in diet at weaning^47^. The abundance of *Bacteroidota* was higher in the PRO/CON group compared with the CON/CON group. The abundance of this phylum has previously been shown to increase PW^48^, and is associated with PW dietary changes. In the present study, its increased abundance is likely linked with the higher abundance of members of the *Prevotellaceae* family (*Prevotella, Alloprevotella* and *Prevotellaceae*) in the PRO/CON group. Members of this family, for example *Prevotella* spp., can break down polysaccharides in the cell wall of cereals using xylanases, mannanases and β-glucanases^49–51^ and are therefore involved in the fermentation of undigested carbohydrates with the resultant production of VFA, thereby contributing to increased energy harvest for the host. Hence, this increased abundance of members of the *Prevotellaceae* family may help to explain the improved feed efficiency during the first 14 days PW in offspring from the probiotic-supplemented sows and the subsequent improvements in growth in these animals reported previously in our parallel publication^13^. Data on the role of *Prevotella* in the pig gut are conflicting but a recent review concluded that studies showing positive associations with feed efficiency and growth outnumber those demonstrating negative correlations^14^. The genera *Blautia* and *Turicibacter*, which belong to the phylum *Firmicutes* were also increased in the PRO/CON group compared to the CON/CON group. *Blautia* are also involved in carbohydrate fermentation and recently, *Turicibacter* was shown to be positively correlated with body weight in pigs^52^. *Blautia* are a member of the *Lachnospiraceae* family that produce butyric acid and are thought to be involved in the alleviation of inflammatory and metabolic diseases and to possess antibacterial activity due to the production of bacteriocins^53,54^. *Blautia* have also been positively associated with feed efficiency in pigs^55^. Several other genera associated with butyric acid production, such as *Ruminococcus* and *Roseburia,* were also increased in the ileal digesta of the PRO/CON group. Butyric acid producing genera often use acetate in the butyryl-coenzyme A: acetate CoA-transferase pathway^55,56^. *Prevotella* spp., which were also increased in abundance and are acetate producers, can facilitate this process^51,57,58^. Thus, the combined increase in acetate and butyric acid producers in the ileal digesta of the PRO/CON group, may have contributed to the higher concentration of butyric acid in the ileal digesta of pigs weaned from the probiotic-supplemented sows. Interestingly, on d118 PW *Lactobacillus* was more abundant in the PRO/CON group compared with the CON/CON and CON/PRO groups. *Lactobacillus* spp. are considered beneficial and are thought to enhance gut health through competitive exclusion of pathogens, antioxidant activity and immunomodulation^59^. Furthermore, *Lactobacillus* is consistently enriched in the large intestine of more feed-efficient pigs^14^. Other studies in which *Bacillus* probiotics have been directly supplemented to weaned and finishing pigs have also shown increased *Lactobacillus*^60,61^.

*Firmicutes* abundance was increased in the PRO/PRO group compared with the CON/CON group in the ileal digesta at d8 PW. This may be considered beneficial, as this phylum is thought to contribute to VFA production and the regulation of systemic immune responses, contributing to energy salvage, the inhibition of opportunistic pathogens and protection against inflammation^62^. Within the *Firmicutes*, *Clostridium sensu stricto* 1 and *Terrisporobacter* were more abundant in the PRO/PRO compared with the CON/CON group. Both of these genera are common components of the ileal microbiota of pigs^63,64^. A significant age-associated increase in *Clostridium sensu stricto* 1 has previously been observed in pigs PW, suggesting it may be associated with increased gut maturation^65^. Shannon α-diversity was reduced in the ileum of pigs in the PRO/PRO group compared with the CON/CON and PRO/CON groups. A high level of bacterial diversity is often associated with ecosystem stability, resistance to pathogen invasion and development of the immune system^66^. Despite these associations, a reduction in diversity is often observed in pigs supplemented with ZnO and regularly associated with improvements in growth performance and parameters of intestinal health^67,68^. The reduced bacterial diversity in the present study was not associated with any negative effects on pig growth or health (in fact, it was the opposite) in this study as reported previously in our parallel publication^13^. Administration of *Bacillus* spores has previously been shown to reduce α-diversity of the faecal microbiota of piglets when compared to antibiotic-supplemented piglets^69^. In another study completed by our group, α-diversity was lower in pigs supplemented with the same *Bacillus* spores as used here for 56 days PW compared with pigs only supplemented for 28 days PW or with pigs supplemented with an antibiotic-ZnO combination^70^. This suggests that the duration of supplementation is an important factor influencing the impact of *Bacillus* spore supplementation on bacterial diversity.

The abundance of the genus *Escherichia/Shigella* was higher in all three probiotic-supplemented groups compared with the CON/CON group. Previously, the same probiotic strain was shown to reduce porcine pathogenic *E. coli *in-vitro^71^. Direct supplementation of this strain to pigs PW also led to a reduction in *E. coli* in the ileum; however, none of the *E. coli* isolates possessed haemolytic activity suggesting they were commensal species^72^. The inconsistency of findings across studies is likely due to the different experimental methods employed i.e. in-vitro vs in-vivo and traditional culturing vs 16S rRNA gene sequencing. The genera *Escherichia* and *Shigella* cannot be distinguished using 16S rRNA gene sequencing and a BLAST search found that the differentially abundant sequence in the current study matches several *E. coli* strains, other *Escherichia* spp. and some *Shigella* spp. Thus, it is not possible to determine which species/serotype is in fact increased. Although the genus *Escherichia/Shigella* has been shown to be more abundant in pigs with diarrhoea^73^, this was in rectal not ileal samples and during suckling, rather than the PW period. In fact, *Escherichia/Shigella* is generally abundant in the ileum of healthy pigs, i.e. up to 23% abundance in some studies^63^ and *Shigella* spp. are not known to cause disease in pigs^74^. One of the main sequence matches in the current study for which the serotype was listed is *E. coli* 0157:H7, which is a food-borne pathogen which causes disease in humans. While pigs may carry this serotype, it is not commonly associated with disease in pigs^75^. The predominant *E. coli* serotype causing PWD in pigs is 0149^76^ and this was not identified in the BLAST search. Furthermore, diarrhoea was not observed in any of the treatment groups in the present study and villous height was improved in the duodenum and ileum of pigs weaned from the probiotic-supplemented sows, as reported in our parallel publication^13^. The growth performance and absence of symptoms in these pigs^13^ would suggest that the sequence detected in this study belongs to a commensal rather than a pathogenic species. If indeed the sequence does represent a commensal species, its increased abundance may reduce the susceptibility of the probiotic-supplemented pigs to colonisation by a pathogenic strain, as strains of the same species are more likely to occupy the same ecological niche than strains of different species^77^. However, it cannot be concluded from the current data that the differentially abundant *Escherichia/Shigella* species/strains would not become toxigenic under the appropriate conditions.

## Conclusion

The novelty of this study lies in the fact that the effects of maternal and PW probiotic supplementation on the microbiota were monitored in the sows’ colostrum and faeces, and in the offspring digesta/faeces from birth through to the finishing period. The data show that maternal supplementation with *B. altitudinis* spores during late gestation and throughout lactation had a greater impact on the intestinal microbiota than direct PW supplementation. These findings confirm the hypothesis that early-life microbiome manipulation can be achieved through maternal supplementation. Offspring microbiome modulation appears to occur in the present study, via manipulation of colostral composition and colostral microbiome, but may also be related to the presence of the probiotic in the sows’ faeces, as reported previously. The offspring of probiotic-supplemented sows had increased abundance of bacterial taxa associated with the breakdown of complex carbohydrates and nutrient utilisation in the ileum during the early PW period. This is potentially a driver of the improved feed efficiency and intestinal morphology observed during this period as already reported in a parallel publication. The increased abundance of *Lactobacillus* during the late PW period in the offspring of probiotic-supplemented sows, which were not supplemented PW, may have contributed to the improved growth observed in this group during the finishing period, and the resultant increase in carcass weight as also reported previously. In conclusion, the findings from the present study, suggest that the early PW gut microbiota of the offspring of *B. altitudinis*-supplemented sows was better suited to the digestion of the PW cereal-based diet, which may help to explain the previously reported improvements in feed efficiency and carcass weight. As pigs were individually housed from d8 PW, further research is required to elucidate if the responses observed will also be seen with commercial group housing practices.

## Materials and methods

The colostrum, faecal and intestinal samples analysed in this study were collected during a previous study conducted by Crespo-Piazuelo et al.^13^. The materials and methods pertaining to experimental design, animal housing and management and probiotic spore preparation are outlined briefly here with, full detail available in the Crespo-Piazuelo et al.^13^ publication.

### Ethical approval

Ethical approval for this study was granted by the Teagasc Animal Ethics Committee (approval no. TAEC148/2017) and the project was authorised by the Health Products Regulatory Authority (project authorisation no. AE19132/P066). The experiment was conducted in accordance with Irish legislation (SI no. 543/2012) and EU Directive 2010/63/EU on the protection of animals used for scientific purposes and in compliance with the ARRIVE guidelines.

### Experimental design and diets

The experiment was designed as a 2 × 2 factorial arrangement. Briefly, on d100 of gestation, 24 sows (Large White × Landrace) were selected and allocated to one of 12 blocks (2 sows per block), by parity (1–4 previous litters), body weight (209–311 kg) and back fat depth (13.5–22 mm) and assigned at random to either: (1) a standard gestation and lactation control diet (CON, *n* = 12), or (2) a standard gestation and lactation diet supplemented with *B. altitudinis* WIT588 spores (~ 4 × 10^9^ spores daily from d100 of gestation to farrowing and ~ 1.2 × 10^10^ spores daily during lactation for 26 days until weaning of litters, administered as outlined below; PRO, *n* = 12). Cross-fostering of piglets was performed between 24 and 48 h *post-partum* to equalize litter size (14 piglets/litter) if necessary, but only within the same treatment group.

At weaning, 144 piglets from these sows (*n* = 72/sow treatment) were selected across all litters, on the basis of mean body weight for the litter (7.27 (SEM 0.168) kg) and balanced for gender. The piglets were blocked by sow treatment, sex, body weight and litter of origin and randomly assigned to dietary treatments. Offspring from both sow treatments were assigned as same gender pairs to either a CON (no probiotic) or PRO (probiotic-supplemented) treatment for 28 days PW, resulting in four treatment groups (*n* = 36 piglets/treatment): (1) piglets weaned from CON sows, fed a CON diet (CON/CON); (2) piglets weaned from CON sows, fed a PRO diet (CON/PRO); (3) piglets weaned from PRO sows, fed a CON diet (PRO/CON); and (4) piglets weaned from PRO sows, fed a PRO diet (PRO/PRO). A sub-set of these pigs were sampled as outlined below. Probiotic supplementation consisted of ~ 1 × 10^9^ *B. altitudinis* WIT588 spores top-dressed onto feed daily, as described previously^13^ and as outlined below. Probiotic supplementation ceased at d28 PW, but samples were collected up to d118 PW (the end of the finishing period).

The ingredient composition and nutrient content of all sow and offspring diets are shown in Supplementary Table S4. Details on diet formulation and feed and water delivery are described by Crespo-Piazuelo et al.^13^.

### Preparation and administration of probiotic spores

*Bacillus altitudinis* WIT588 is a rifampicin resistant variant of *B. altitudinis* NCIMB 43558 (WIT572), a seaweed-derived isolate characterized both in vitro and in vivo as a probiotic for pigs, which was used to facilitate enumeration in the porcine GIT^13,71,72,78^. The strain was first referred to as *Bacillus pumilus* on the basis of sequencing of the *gyrB* and *pyrE* genes^72^, but has since been identified as *B. altitudinis* on the basis of whole genome sequencing (unpublished data). The *B. altitudinis* WIT588 spores were prepared as described previously by Prieto et al.^72^, suspended in sterile water, aliquoted and stored at − 20 °C until use. On the morning of administration, the thawed spore suspensions were diluted in distilled water to the required dose and top-dressed onto the feed as described by Crespo-Piazuelo et al.^13^.

### Animal housing and management

Sow and offspring housing and management is described in detail by Crespo-Piazuelo et al.^13^. Briefly, PRO sows were housed separately from CON sows in order to minimise cross contamination. Farrowing pens (2.5 m × 1.8 m) had a farrowing crate on a partially slatted floor with a heated floor pad for piglets. The temperature of the farrowing rooms was automatically controlled and was maintained at ~ 24 °C at farrowing and gradually reduced to 21 °C by d7 of lactation. At weaning, piglets were housed in same sex pairs in 72 pens (*n* = 2 pigs/pen) across 4 rooms, with treatments distributed equally across rooms. Pens (1.2 m × 0.9 m) were fully slatted with plastic flooring (Faroex, Manitoba, Canada). Empty pens were left between treatments to prevent probiotic cross-contamination. On d8 PW, 40 pigs (*n* = 10/treatment) were sacrificed by captive bolt stunning followed by exsanguination to facilitate sampling of digesta. At the same time, one pig from each of the remaining pens (32 pens) was removed from the trial and the remaining piglets (*n* = 72) were individually penned until d55 PW. The temperature of the weaner rooms was maintained at 28 °C for the first 7 days PW, gradually reduced to 22 °C by d28 PW and maintained at 22 °C until d56 PW. At d56 PW, pigs were moved to one of four finisher rooms, where they were individually penned in fully slatted pens (1.81 m × 1.18 m) until the end of the experimental period (d118 PW). Air temperature was maintained at 20 to 22 °C.

### Sampling

During sampling of sows and offspring, strict hygiene measures were taken to prevent cross-contamination between treatments as follows. Pigs not receiving probiotic were handled first, followed by the probiotic treatment groups. Gloves were changed between pigs, and fresh disposable overalls were worn by all personnel and changed prior to commencing sampling of each treatment group. All equipment used was disinfected thoroughly with 1% Virkon^®^ after use to prevent cross contamination at subsequent samplings.

### Colostrum sampling

Colostrum samples (*n* = 12 sows/treatment) were collected by manual milking of the first four teats immediately distal to the sow’s head on one side of the udder within 4 h of farrowing. Iodine was used to thoroughly clean the area surrounding the teats prior to sample collection and the first 1 ml of colostrum was discarded. Samples (2 × 4.5 ml in sterile tubes) were snap frozen in liquid nitrogen and stored at − 80 °C until analysis.

### Faecal sampling

Faecal samples were collected from sows (*n* = 24) directly from the rectum using gentle digital stimulation on d100 of gestation, at farrowing, on d13 of lactation and at weaning of litters (~ d26 of lactation). Pre-weaning, rectal swabs were taken from offspring on ~ d13 of lactation (*n* = 12 pig replicates per treatment) using sterile viscose tip swabs (Sarstedt Ltd, Drinagh, Wexford, Ireland), faecal samples were obtained by digital rectal stimulation at weaning, d13 PW, d28 PW and d56 PW from the same pigs (*n* = 10 pig replicates per treatment) and freshly voided faecal samples were collected on d118 PW (*n* = 5 for CON/CON, *n* = 4 for CON/PRO, *n* = 4 for PRO/CON and *n* = 6 for PRO/PRO, respectively). Rectal swabs were put on ice prior to storage at − 80 °C and faecal samples were collected into sterile tubes and immediately snap frozen in liquid Nitrogen and stored at − 80 °C until analysis.

### Intestinal sampling

Forty piglets (*n* = 10 pigs per treatment), were humanely sacrificed on d8 PW by captive bolt stunning followed by exsanguination and the entire intestinal tract was immediately removed. Digesta samples from the ileum (15 cm proximal to the ileo-caecal junction), caecum (terminal tip) and rectum were collected aseptically into sterile tubes, snap frozen in liquid Nitrogen and stored at − 80 °C until analysis.

### DNA extraction from colostrum, rectal swabs, digesta and faecal samples

Total DNA was extracted from rectal swabs and faecal and digesta samples using the QIAamp DNA stool minikit (Qiagen, Crawley, United Kingdom) according to the manufacturer’s instructions, apart from adding a bead beating step and increasing the lysis temperature to 95 °C, to increase DNA yield^79^. The volume of buffer ATE was also reduced from 200 to 30 µl and it was allowed to sit on the filter membrane of the spin column for 5 min prior to centrifugation (both to maximise DNA concentration). For extraction from rectal swabs, 1 ml of sterile phosphate buffered saline was added to each swab and vortexed for 2 min. The resultant liquid was then transferred to a sterile 1.5 ml tube and centrifuged for 2 min at 14,000 rpm. The supernatant was discarded and the remaining pellet was used for DNA extraction. For the faecal and digesta samples, 0.25 g of each sample was used for the extractions.

Total DNA was extracted from colostrum samples using the DNeasy PowerFood Microbial Kit (Qiagen). The manufacturer’s instructions were followed with some modifications as follows: (1) The initial homogenization step was omitted; (2) 1.8 ml of colostrum was added to the 2 ml collection tube in triplicate in order to process 5.4 ml colostrum in total; (3) the duration of the first centrifugation step was increased to 15 min to maximise pellet formation; (4) the re-suspended cells were added to a screw-capped tube containing autoclaved zirconia beads (0.125 g of 0.1 mm and 0.125 g of 1.0 mm, a single bead of 2.5 mm; Stratech Scientific, Ely, UK) and bead beating was performed in place of vortexing in PowerBead tubes for 3 min followed by 1 min rest and then for 2 min followed by 1 min rest, to prevent DNA shearing; (5) in the final step, the volume of elution buffer was reduced from 100 to 20 µl and it was allowed to sit on the filter membrane of the spin column for 5 min prior to centrifugation to maximise DNA concentration.

### 16S rRNA gene sequencing of colostrum, digesta and faecal microbiota

Microbial profiling was performed using high-throughput sequencing of the V3–V4 region of the 16S rRNA gene (paired-end reads of 2 × 300 bp) following 600 amplification cycles on an Illumina MiSeq platform. The Illumina-recommended 16S metagenomic library preparation (Nextera) protocol was followed. Paired-end reads in all samples were quality assessed using FastQC (v0.11.7)^80^ and quality trimmed (cuttoff-phred = 20) using BBduk from the BBTools suite (https://jgi.doe.gov/data-and-tools/bbtools/). Primers and low quality read tails were also removed at this step. The DADA2 pipeline^81^ was used to perform read filtering and de-replication, chimera detection and removal, read-pair merging and inference of amplicon sequence variants (ASV) in each sample. Taxonomy was assigned to each derived ASV using a naive Bayesian classifier method against the SILVA database (Version 128)^82^. Species level classification was identified, where possible using taxonomic assignment for exact sequence matches to the SILVA database or by blasting the sequences against the nucleotide database of the U.S. National Center for Biotechnology Information (NCBI) (available at: https://blast.ncbi.nlm.nih.gov/Blast.cgi). Alpha diversity index (Shannon) and β-diversity (UniFrac) analyses were calculated using the phyloseq package^83^ in R version 4.02^84^. nMDS and MDS plots based on the UniFrac distance matrix were performed and subsequently plotted using the ggplot2 package^85^ in R version 4.02^84^.

### Short chain fatty acid analysis of ileal digesta

Short chain fatty acid profiles of the ileal digesta were determined by Alimetrics Diagnostics (Espoo, Finland) using gas chromatography (GC; Agilent Technologies, Santa Clara, CA, USA) with pivalic acid (Sigma-Aldrich, St. Louis, MO, USA) used as an internal standard. The chromatography procedure used a glass column packed with 80/120 Carbopack B-DA/4% Carbowax stationary phase, helium as a carrier gas, and a flame ionization detector (FID) and has been described previously by Apajalahti et al.^86^. Lactic acid and the VFA (acetic acid, propionic acid, isobutyric acid, butyric acid, 2-methylbutyric acid, isovaleric acid and valeric acid) were derivatised to the respective phenyl esters using phenyl chloroformate reagent. The resulting esters were analysed by Agilent GC-FID (Agilent Technologies). Matrix-matched internal standard calibration with butyric-d7- and acetic-d3 acids was used in quantitation.

### Statistical analysis

Statistical analysis of OTU abundance was performed using DeSeq2^87^ in R version 4.02^84^. Low abundance ASVs were manually filtered and ASVs were retained if the sum of reads for that ASV across all samples was greater than 0.005% of all ASVs. A false discovery rate of < 0.05 was indicative of significant abundance difference between groups. For each taxon, differences between the median abundances of samples in each treatment group were assessed using the Wilcoxon Rank Sum test of the R package Metacoder^88^. Weighted UniFrac distance matrices were subjected to Permutational ANOVA to test for significant effects of treatments on the microbial community structure in samples at various time-points. Results of all analyses are presented using adjusted *P*-values.

For statistical analysis of the ileal SCFA results, the data were first investigated using the Shapiro–Wilk and Kolmogorov–Smirnov tests within the UNIVARIATE procedure of SAS 9.4 (SAS Institute Inc., Cary, NC). As the data were not normally distributed, they were further analysed using the GLIMMIX procedure of SAS with the log link function and a gamma distribution. The ilink function was used to back-transform the data to the original scale. The model included the effects of maternal and PW treatment as fixed effects and their associated interaction. The individual pig was the experimental unit. The least square means were compared between the treatment groups using the *t* test and the *P*-values were corrected for multiple comparisons using the Tukey adjustment. The Satterthwaite method was used to estimate the degrees of freedom. The data are presented in the text and tables as the least square means with the pooled standard errors of the mean. The probability level that denoted significance was *P* ≤ 0.05, while 0.05 < *P* < 0.10 was considered a numerical tendency.

## Supplementary Information

Supplementary Information.

## Data Availability

The raw sequencing data generated by this study were deposited in the NCBI Sequence Read Archive (SRA) under BioProject accession number PRJNA878669.
