# Supplementary material for: Maternal and/or post-weaning supplementation with Bacillus altitudinis spores modulates the microbial composition of colostrum, digesta and faeces in pigs
Source: Sci Rep. 2023 Jun 1;13:8900. doi: 10.1038/s41598-023-33175-2 (PMC10233552; doi:10.1038/s41598-023-33175-2)
Supplement: Supplementary file 1 — Supplementary Information. [file 41598_2023_33175_MOESM1_ESM.docx]

**Maternal and/or post-weaning** **supplementation with *Bacillus* *altitudinis* spores modulates the microbial composition of colostrum, digesta and faeces in pigs**

Ruth Rattigan^1^, Peadar G. Lawlor^2^, Paul Cormican^3^, Daniel Crespo-Piazuelo^2^, James Cullen^1^, John P. Phelan^1^, Samir Ranjitkar^2^, Fiona Crispie^4, 5^ and Gillian E. Gardiner^1*^

^1^Eco-Innovation Research Centre, Department of Science, Waterford Campus, South East Technological University, Waterford, Ireland

^2^Pig Development Department, Animal and Grassland Research and Innovation Centre, Teagasc, Moorepark, Fermoy, Co. Cork, Ireland

^3^Animal & Grassland Research and Innovation Centre, Teagasc, Grange, Dunsany, Co. Meath, Ireland

^4^APC Microbiome Ireland, University College Cork, Cork, Ireland.

^5^Food Research Centre, Teagasc, Moorepark, Fermoy, Cork, Ireland

*Corresponding author: Gillian Gardiner, +353 51 302626, ggardiner@wit.ie

| 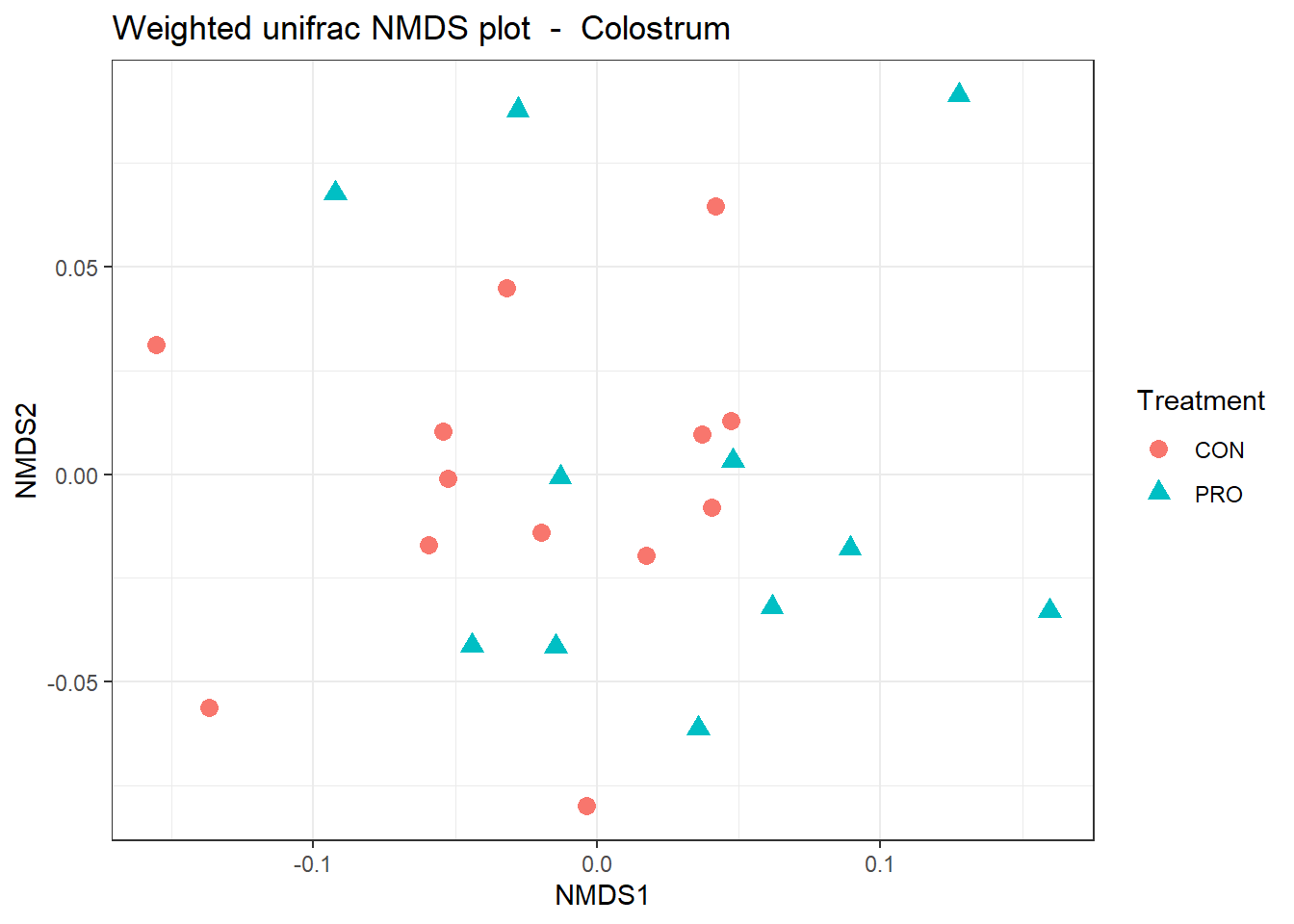 |
| --- |

**Supplementary Figure S1.** The effect of sow treatment on unweighted UniFrac distance (β-diversity) of the microbiota within the sow colostrum. Maternal treatment: CON, control; PRO, probiotic

| **A) Mean relative abundance of bacterial phyla**  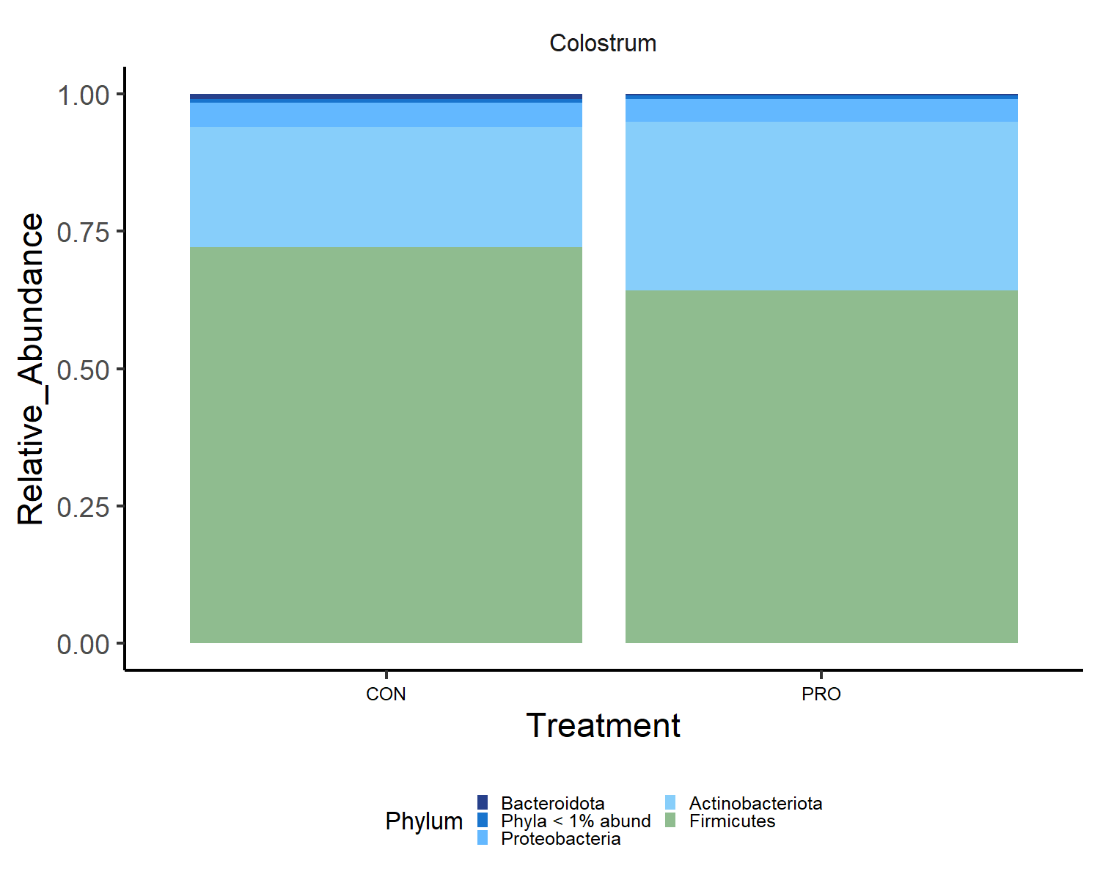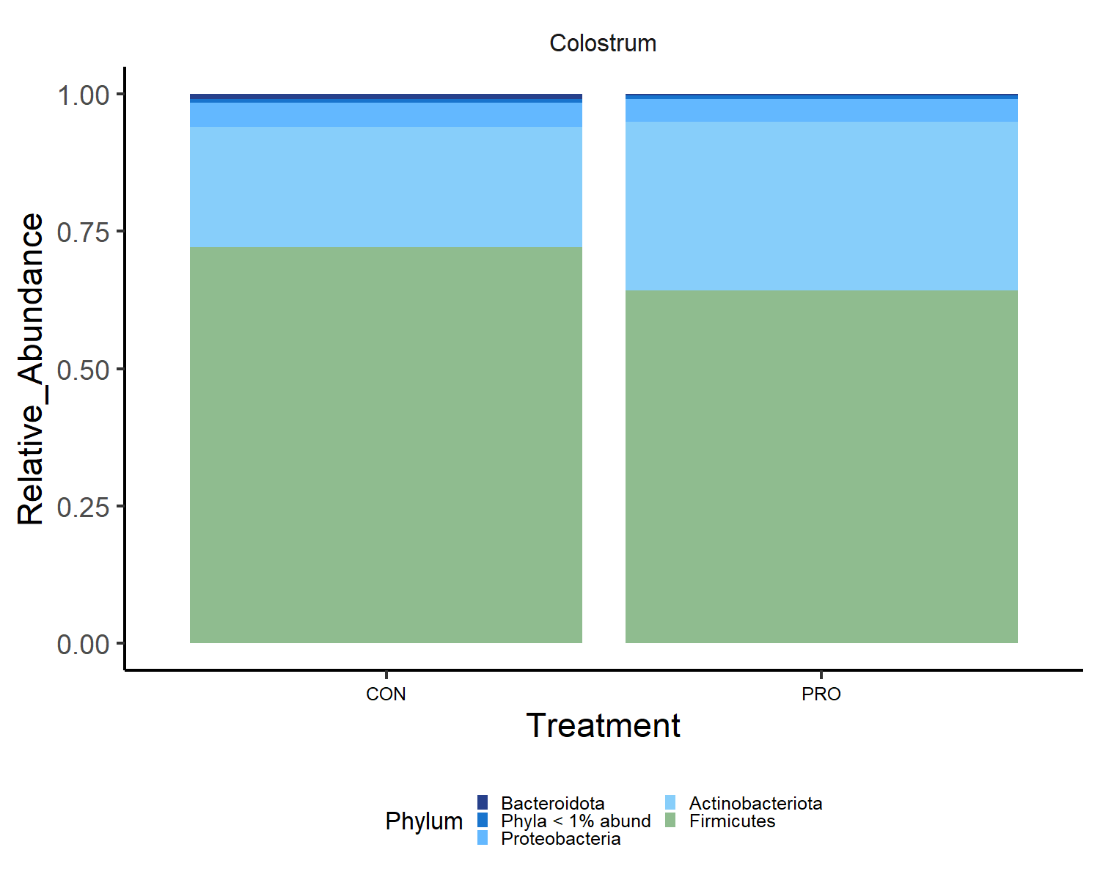 |
| --- |
| **B) Mean relative abundance of bacterial genera**  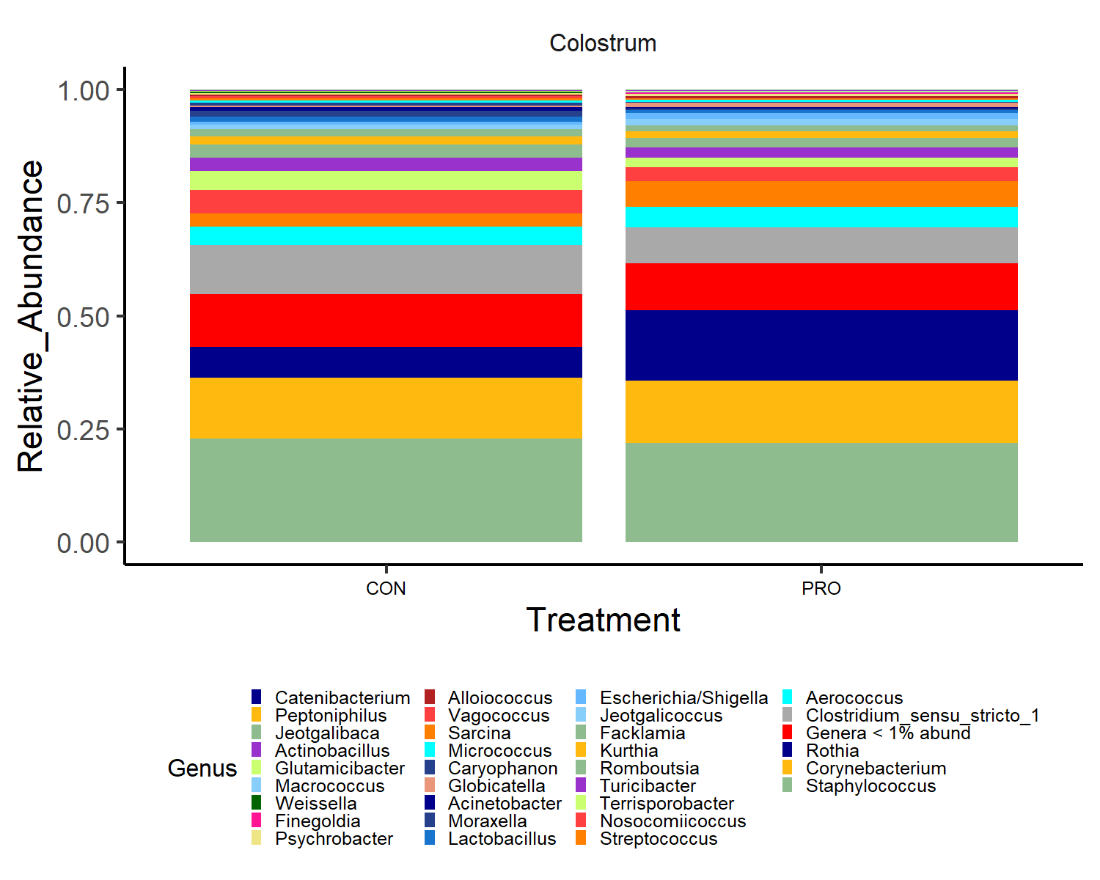  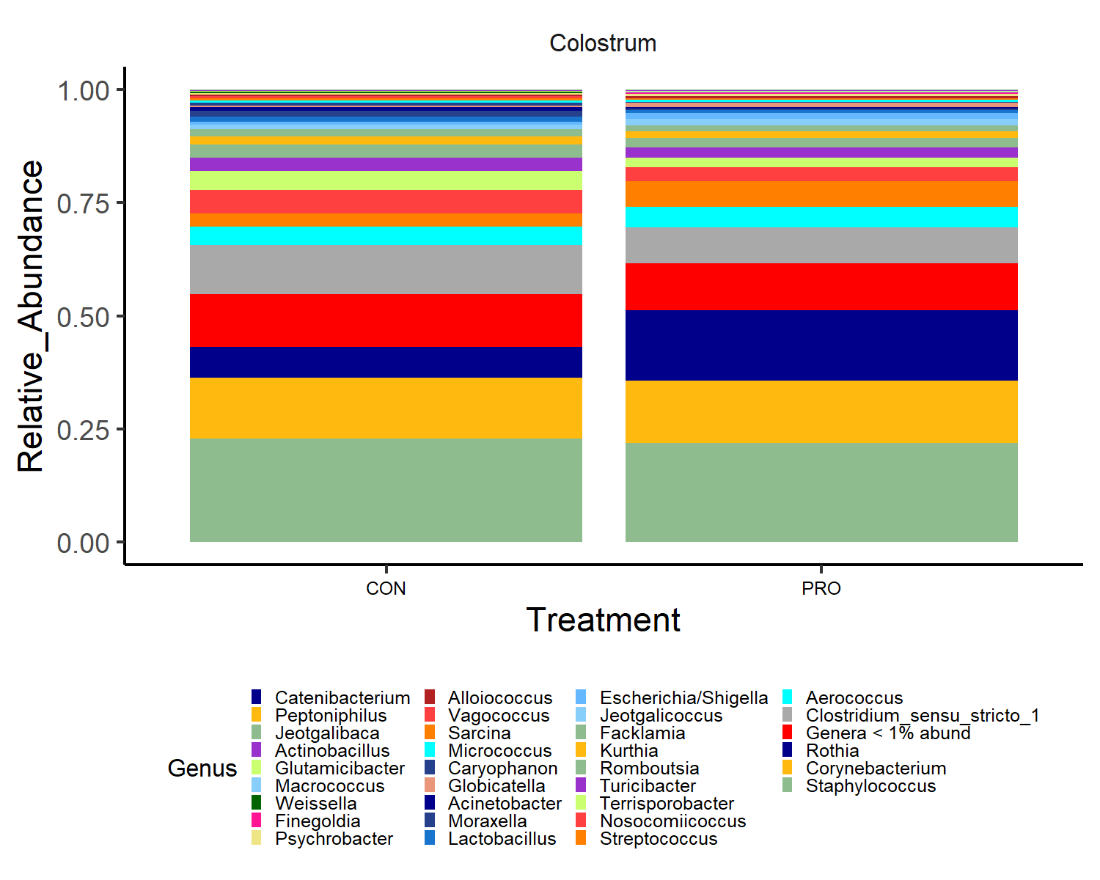 |

**Supplementary Figure S2.** The effect of treatment on the mean relative abundance of bacterial phyla (A) and bacterial genera (B) within the sow colostrum.

Maternal treatment: CON, control; PRO, probiotic.

| 1. **Shannon α-diversity**   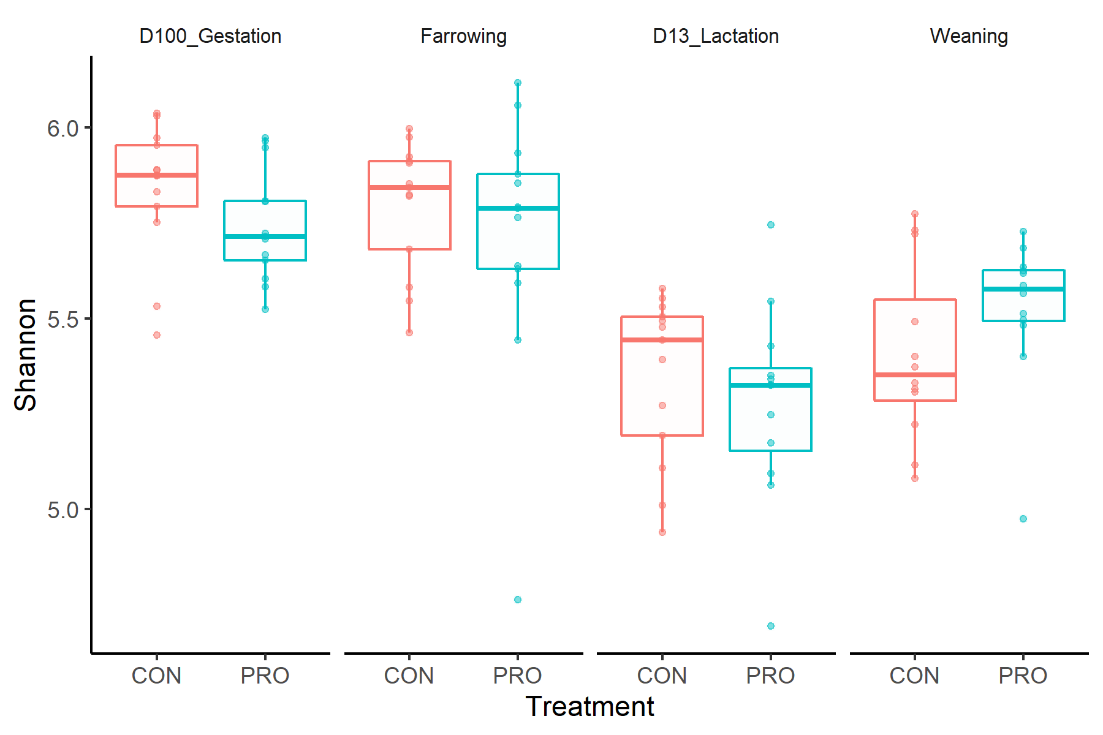 |
| --- |
| 1. **MDS plot of the unweighted UniFrac distance**   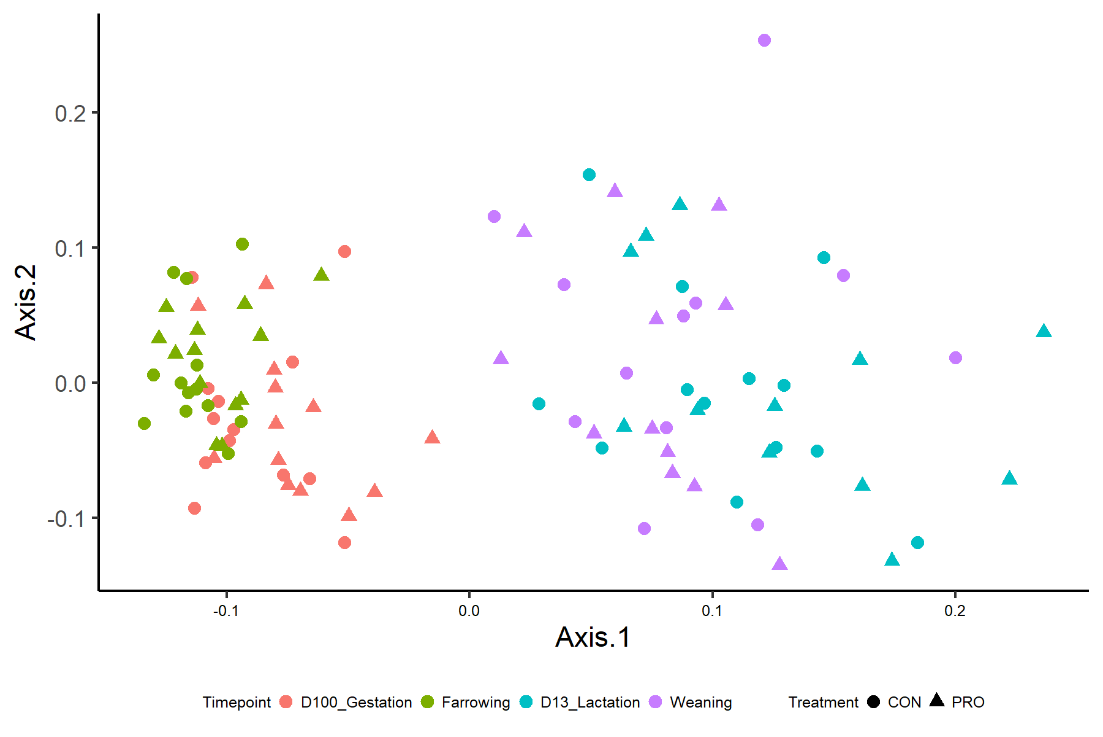  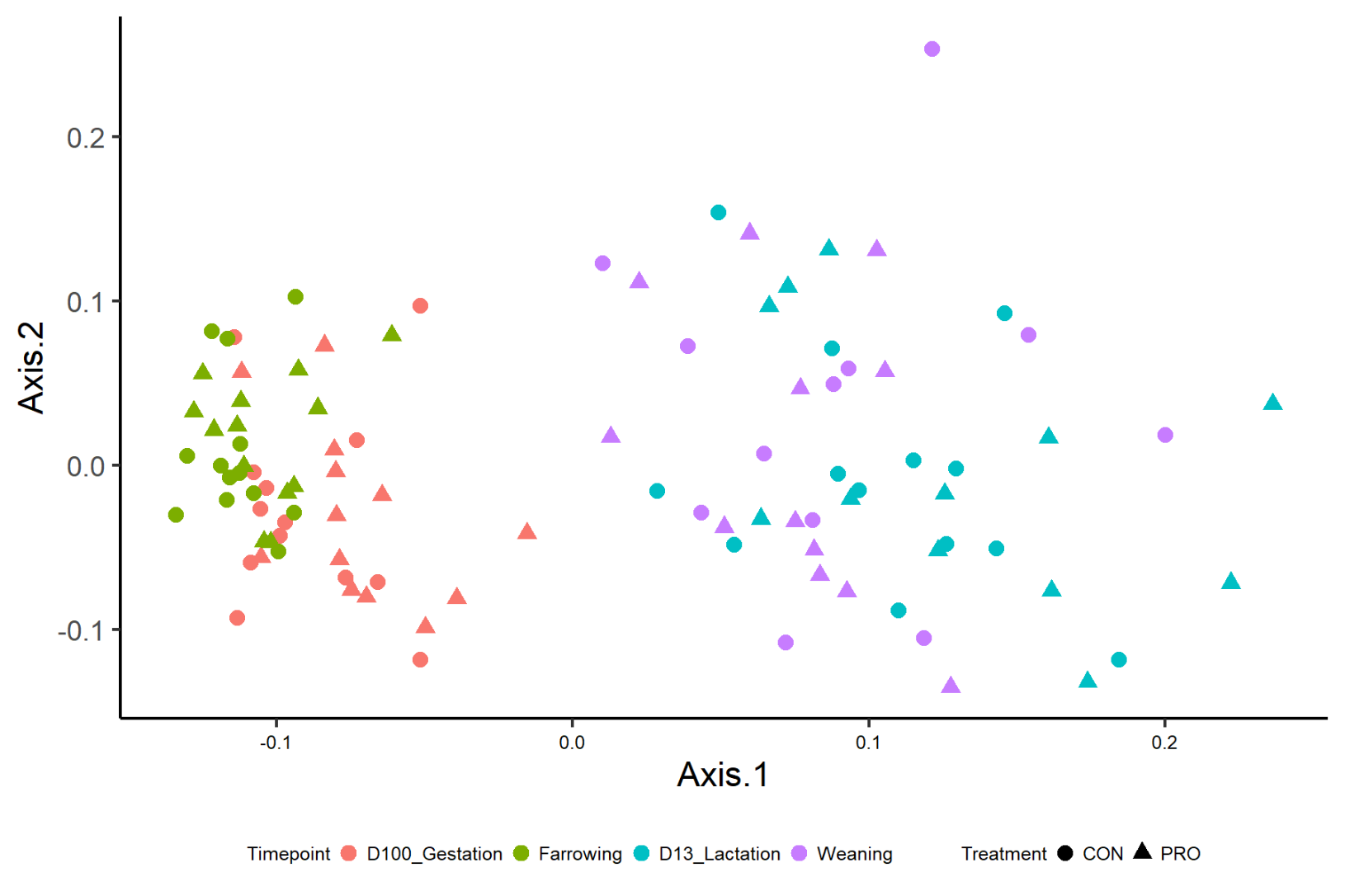 |

**Supplementary Figure S3.** The effect of treatment on Shannon α-diversity (A) and the unweighted UniFrac distance (β-diversity) (B) of the microbiota within the sow faeces at all time-points.

Maternal treatment: CON, control; PRO, probiotic. MDS, multidimensional scaling.

| 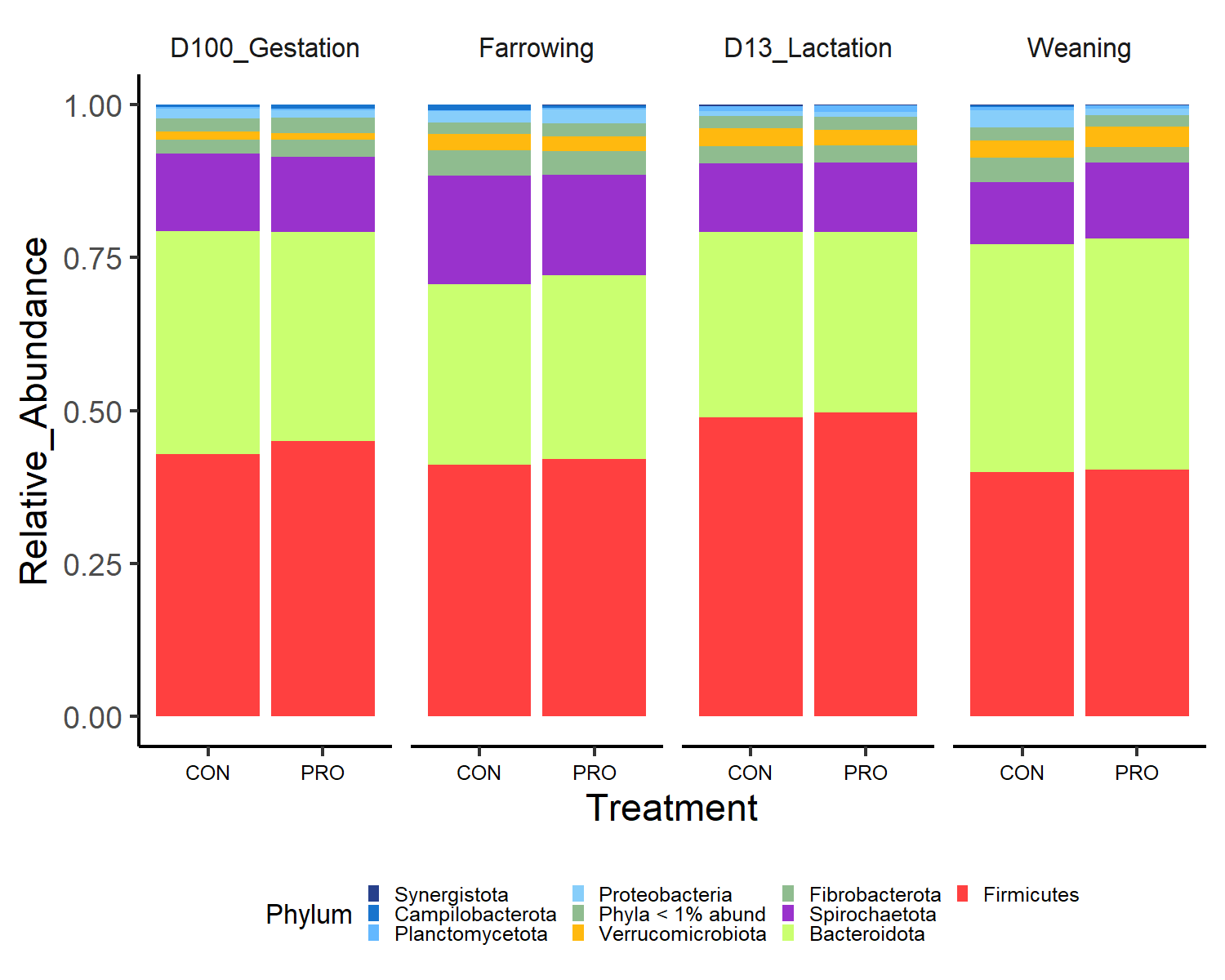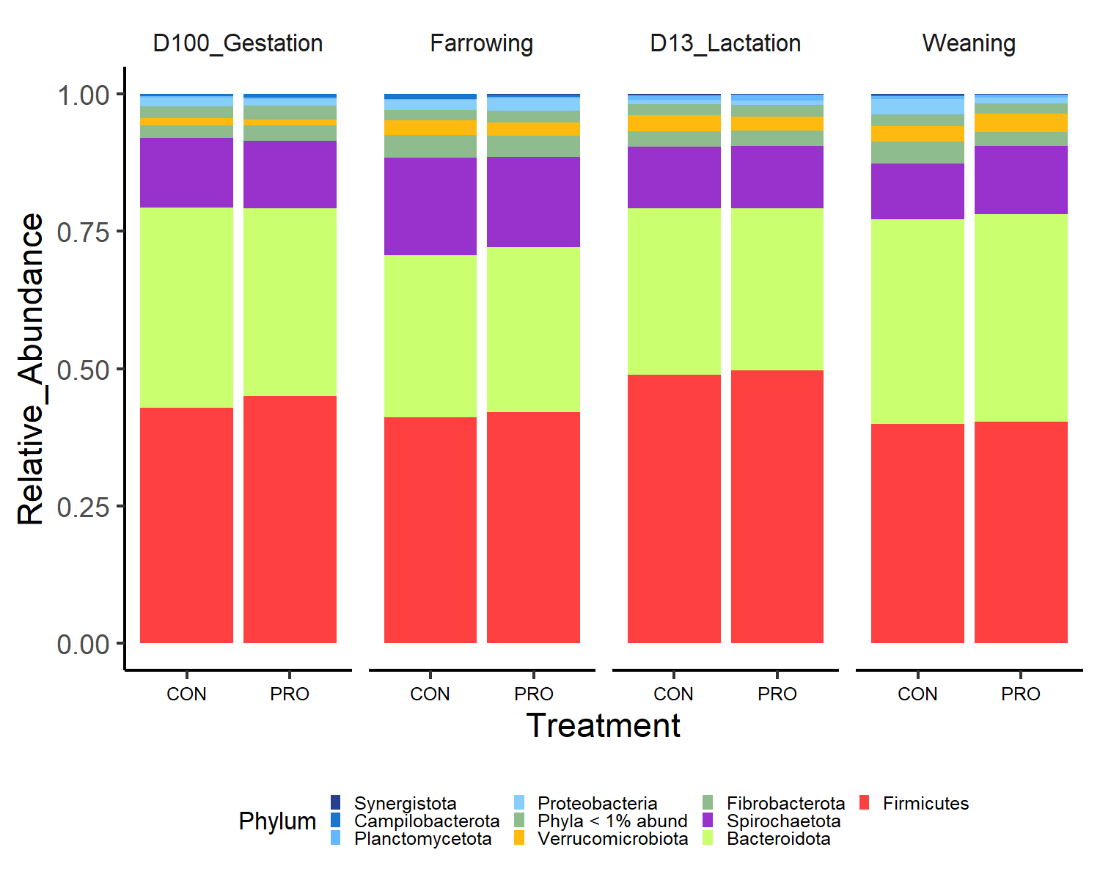 |
| --- |

**Supplementary Figure S4.** Mean relative abundance of bacterial phyla in the sow faeces at all time-points. Maternal treatment: CON, control; PRO, probiotic

| 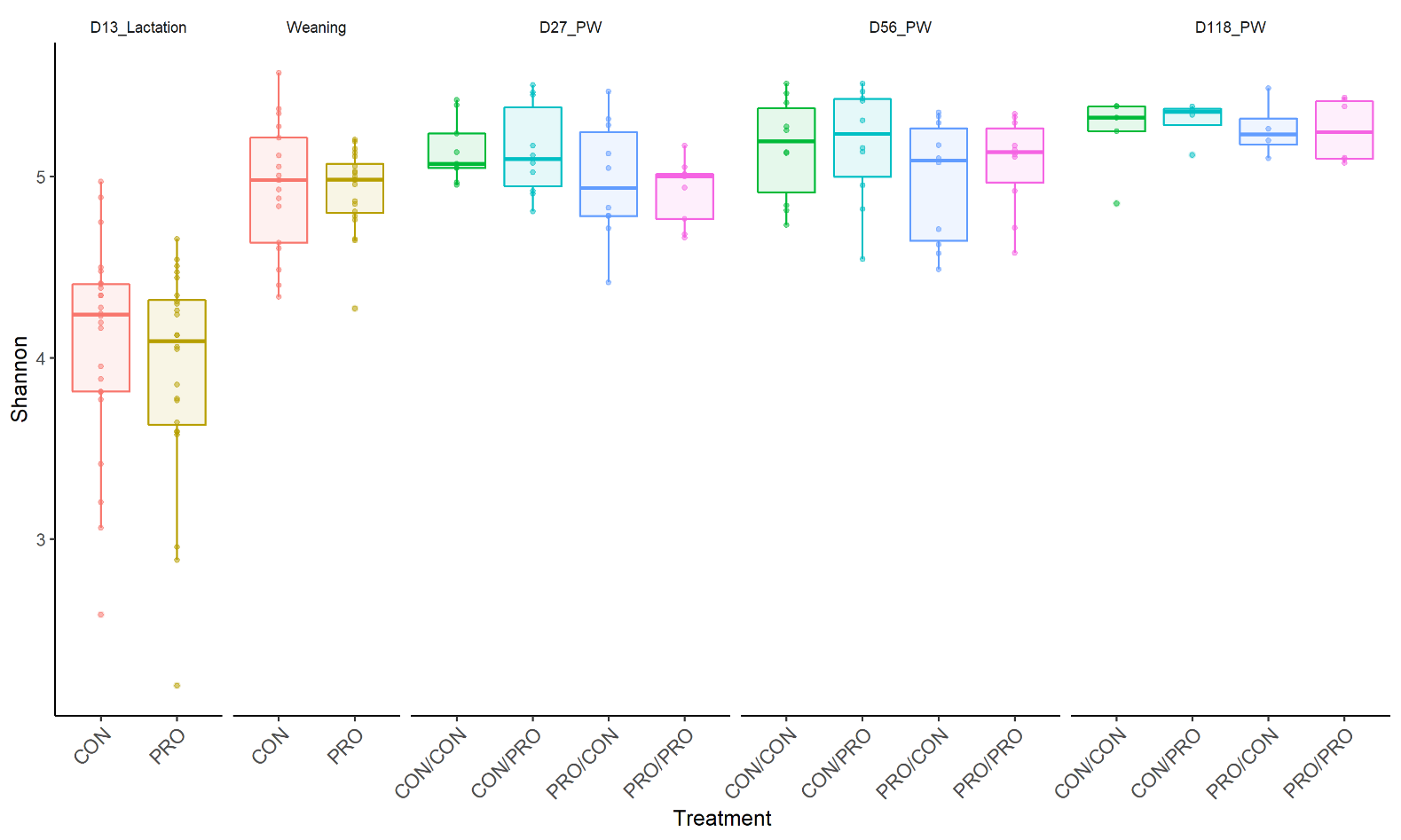  Weaning  D118 PW  D56 PW  D27 PW  D13 lactation |
| --- |

**Supplementary Figure S5.** Effect of maternal and post-weaning treatment on Shannon α-diversity in offspring faeces at all sampling points. Treatments are as follows (maternal treatment/post-weaning treatment): CON/CON, CON/PRO, PRO/CON and PRO/PRO; where CON = control and PRO = probiotic. No differences in Shannon α-diversity were identified between treatments across sampling points (*P* > 0.05).

**Supplementary Table S1. Differentially abundant bacterial taxa within the colostrum of sows in the control and probiotic-supplemented groups.**

| **Taxa** | **Maternal Treatment** | | **SEM^1^** | ***P* value** |
| --- | --- | --- | --- | --- |
|  | **CON** | **PRO** |  |  |
| *P- Actinobacteriota* | 21.94 | 30.73 | 1.940 | 0.025 |
| *F- Vagococcaceae* | 0.513 | 0.011 | 0.2114 | 0.013 |
| *F- Micrococcaceae* | 7.750 | 16.183 | 1.8534 | 0.020 |
| *F- Aerococcaceae* | 6.158 | 7.325 | 0.7284 | 0.025 |
| *G- Rothia* | 6.92 | 15.74 | 2.431 | 0.015 |
| *G- Globicatella* | 0.53 | 1.22 | 0.246 | 0.001 |
| *G- Vagococcus* | 0.50 | 0.01 | 0.198 | 0.001 |

CON, control; PRO, probiotic; SEM, standard error of the mean; P, phylum; F, family; G, genus.

^1^Pooled standard error of the mean. Statistical analysis of OTU abundance was performed using DeSeq2^26^ in R version 4.02^24^, where low abundance ASVs were manually filtered and a false discovery rate of < 0.05 was indicative of significant abundance difference between groups. For each taxon, differences between the median abundances of samples in each treatment group were assessed using the Wilcoxon Rank Sum test of the R package Metacoder^27^.

**Supplementary Table S2. Differentially abundant bacterial taxa within the faeces of offspring from control and probiotic-supplemented sows on day 13 of lactation and at weaning (day 26 of lactation).**

| **Taxa** | **Maternal Treatment** | | **SEM^1^** | ***P* value** |
| --- | --- | --- | --- | --- |
|  | **CON** | **PRO** |  |  |
| ***Lactation d13*** |  |  |  |  |
| G- *Allistipes* | 0.046 | 0.013 | 0.0115 | <0.001 |
| ***Weaning (lactation d26)*** |  |  |  |  |
| P- *Synergistota* | 2.608 | 0.763 | 0.4242 | 0.049 |
| F- *Rikenellaceae* | 6.614 | 3.354 | 0.4376 | <0.001 |
| F- *Campylobacteriaceae* | 0.976 | 0.640 | 0.2416 | 0.031 |
| G- *Lachnospiraceae_NK4A136_group* | 0.490 | 2.083 | 0.4483 | 0.013 |
| G- *Rikenellaceae_dgA-11_gut_group* | 2.049 | 0.215 | 0.3399 | <0.0001 |
| G- *Prevotellaceae_UCG-003* | 0.712 | 0.153 | 0.1135 | <0.0001 |
| G- *Muribaculaceae_CAG-873* | 0.621 | 0.126 | 0.1010 | 0.013 |
| G- *Alloprevotella spp.* | 2.496 | 1.240 | 0.3465 | 0.026 |

CON, control; PRO, probiotic; SEM, standard error of the mean; d, day; P, phylum; F, family; G, genus. ^1^Pooled standard error of the mean. Statistical analysis of OTU abundance was performed using DeSeq2^26^ in R version 4.02^24^, where low abundance ASVs were manually filtered and a false discovery rate of < 0.05 was indicative of significant abundance difference between groups. For each taxon, differences between the median abundances of samples in each treatment group were assessed using the Wilcoxon Rank Sum test of the R package Metacoder^27^.

**Supplementary Table S3. Differentially abundant bacterial taxa within the digesta and faeces of pigs post-weaning across the four treatment groups.**

| **Taxa** | **Treatment** | | | | **SEM^1^** | **Contrasts - *P* values** | | | | | |
| --- | --- | --- | --- | --- | --- | --- | --- | --- | --- | --- | --- |
|  | **CON/CON 1** | **CON/PRO 2** | **PRO/CON 3** | **PRO/PRO**  **4** |  | **1 vs 2** | **1 vs 3** | **1 vs 4** | **2 vs 3** | **2 vs 4** | **3 vs 4** |
| **Ileum d8 PW** | | | | | | | | | | | |
| *P- Actinobacteria* | 2.917 | 0.878 | 0.709 | 0.611 | 0.2340 | 0.004 | 0.003 |  |  |  |  |
| *P- Parescibacteria* | 0.337 | 0.031 | 0.071 | 0.012 | 0.0600 | 0.014 |  | 0.003 |  |  |  |
| *P- Bacteroidota* | 4.401 | 8.032 | 13.780 | 2.021 | 1.2664 |  | 0.006 |  |  | 0.004 | 0.001 |
| *P- Firmicutes* | 47.703 | 49.601 | 47.590 | 57.695 | 3.0284 |  |  | 0.017 |  |  |  |
| *P- Fusobacteria* | 0.179 | 0.082 | 0.095 | 0.033 | 0.0280 |  |  | 0.004 |  |  |  |
| *P- Spirochaetota* | 0.099 | 0.842 | 1.057 | 0.021 | 0.1744 |  | 0.018 |  |  |  | 0.0003 |
| *F- Bacillaceae* | 0.007 | 0.866 | 0.041 | 0.391 | 0.0915 | <0.001 |  | <0.001 |  |  |  |
| *F- Weeksellaceae* | 1.398 | 0.047 | 0.034 | 0.050 | 0.2567 | 0.010 | 0.002 | 0.002 |  |  |  |
| *F- Oscillospiraceae* | 0.110 | 0.862 | 1.329 | 0.107 | 0.1924 | 0.012 | 0.002 |  |  |  |  |
| *F-Rikenellaceae* | 0.174 | 1.008 | 1.318 | 0.135 | 0.1857 | 0.016 | 0.002 |  |  |  |  |
| *F- Neisseriaceae* | 0.862 | 0.212 | 0.360 | 0.085 | 0.1472 | 0.024 |  | 0.002 |  |  |  |
| *F- Rhizobiaceae* | 0.468 | 0.018 | 0.022 | 0.013 | 0.0530 | 0.024 | 0.016 | 0.001 |  |  |  |
| *F- Paludibacteraceae* | 0.025 | 0.183 | 0.235 | 0.009 | 0.0368 | 0.024 |  |  |  |  |  |
| *F- Spirochaetaceae* | 0.099 | 0.842 | 1.057 | 0.021 | 0.1744 | 0.024 | 0.003 |  |  |  |  |
| *F- Erysipelotrichaceae* | 0.942 | 2.169 | 5.630 | 2.173 | 0.6850 |  | 0.002 |  |  |  |  |
| *F- Erysipelatoclostridiaceae* | 0.176 | 0.286 | 1.267 | 0.164 | 0.1690 |  | 0.002 |  |  |  |  |
| *F- Acidaminococcaceae* | 0.187 | 0.303 | 0.923 | 0.075 | 0.0844 |  | 0.002 |  |  |  |  |
| *F- Ruminococcaceae* | 0.196 | 0.676 | 1.620 | 0.645 | 0.1672 |  | 0.002 | 0.015 |  |  |  |
| *F- Butyricicoccaceae* | 0.173 | 0.328 | 0.887 | 0.355 | 0.1112 |  | 0.002 |  |  |  |  |
| *F- Selemonadaceae* | 0.121 | 0.226 | 0.715 | 0.115 | 0.0745 |  | 0.002 |  |  |  |  |
| *F- Prevotellaceae* | 1.944 | 5.194 | 8.823 | 0.778 | 0.8905 |  | 0.002 |  |  |  |  |
| *F- Muribaculaceae* | 0.248 | 0.548 | 0.988 | 0.193 | 0.1122 |  | 0.005 |  |  |  |  |
| *F- Tannerellaceae* | 0.033 | 0.128 | 0.393 | 0.111 | 0.0418 |  | 0.006 |  |  |  |  |
| *F- Coriobacteriaceae* | 0.035 | 0.054 | 0.142 | 0.130 | 0.0319 |  | 0.007 |  |  |  |  |
| *Cont.* |  |  |  |  |  |  |  |  |  |  |  |
| **Taxa** | **CON/CON**  **1** | **CON/PRO**  **2** | **PRO/CON**  **3** | **PRO/PRO**  **4** | **SEM^1^** | **Contrasts - *P* values** | | | | | |
|  |  |  |  |  |  | **1 vs 2** | **1 vs 3** | **1 vs 4** | **2 vs 3** | **2 vs 4** | **3 vs 4** |
| *F- Christenellaceae* | 0.065 | 0.341 | 0.702 | 0.127 | 0.1130 |  | 0.008 |  |  |  |  |
| *F- p-2534-18B5-gut group* | 0.040 | 0.173 | 0.219 | 0.040 | 0.0323 |  | 0.011 |  |  |  |  |
| *F- Enterobacteriaceae* | 13.683 | 28.644 | 21.544 | 21.128 | 2.9604 |  | 0.014 | 0.012 |  |  |  |
| *F- Succinivibrionaceae* | 0.055 | 0.162 | 0.312 | 0.021 | 0.0502 |  | 0.014 |  |  |  |  |
| *F- Corynebacteriaceae* | 0.292 | 0.067 | 0.042 | 0.039 | 0.0328 |  | 0.014 | 0.002 |  |  |  |
| *F- Micrococcaceae* | 2.169 | 0.635 | 0.396 | 0.332 | 0.1876 |  | 0.019 | 0.028 |  |  |  |
| *F- Moraxellaceae* | 3.737 | 1.203 | 0.857 | 1.246 | 0.3645 |  | 0.025 |  |  |  |  |
| *F- Campylobacteraceae* | 0.114 | 0.132 | 0.604 | 0.068 | 0.1342 |  | 0.029 |  |  |  |  |
| *F- Anaerovoraceae* | 0.017 | 0.063 | 0.132 | 0.027 | 0.0199 |  | 0.029 |  |  |  |  |
| *F- Lachnospiraceae* | 1.454 | 1.713 | 5.469 | 2.935 | 0.6933 |  | 0.031 |  |  |  |  |
| *F- Mitochondria* | 0.242 | 0.038 | 0.034 | 0.042 | 0.0297 |  | 0.042 |  |  |  |  |
| *F- Peptostreptococcaceae* | 3.928 | 4.874 | 6.898 | 8.190 | 1.3918 |  |  | 0.015 |  |  |  |
| *F- Peptostreptococcales-Tissierelles-fa* | 0.068 | 0.042 | 0.033 | 0.002 | 0.0079 |  |  | <0.001 |  |  |  |
| *F- Clostridiaceae* | 6.673 | 7.344 | 4.527 | 12.616 | 1.6062 |  |  | 0.002 |  |  |  |
| *F- Alcaligenaceae* | 2.194 | 0.430 | 0.867 | 0.181 | 0.2786 |  |  | 0.015 |  |  |  |
| *G- Bacillus* | 0.007 | 0.866 | 0.041 | 0.391 | 0.0915 | <0.001 |  | 0.002 |  |  |  |
| *G- Chryseobacterium* | 1.340 | 0.029 | 0.025 | 0.016 | 0.2544 | 0.002 | 0.013 | <0.001 |  |  |  |
| *G- Escherichia/shigella* | 3.033 | 20.517 | 6.806 | 12.801 | 2.4862 | 0.004 | 0.007 | <0.001 |  |  |  |
| *G- Prevotellaceae_UCG-001* | 0.026 | 0.206 | 0.250 | 0.042 | 0.0410 | 0.015 | 0.009 |  |  |  |  |
| *G- Brevundimonas* | 0.076 | 0.004 | 0.028 | 0.003 | 0.0103 | 0.021 |  |  |  |  |  |
| *G- Pelistega* | 2.194 | 0.430 | 0.867 | 0.181 | 0.2786 | 0.044 |  |  |  |  |  |
| *G- Neisseria* | 0.812 | 0.136 | 0.250 | 0.042 | 0.1016 | 0.015 |  | 0.011 |  |  |  |
| *G- Rikenellaceae_RC9_gut_group* | 0.153 | 0.925 | 1.181 | 0.131 | 0.1689 | 0.044 | 0.004 |  |  |  |  |
| *G- Veillonella* | 0.743 | 0.149 | 0.421 | 0.111 | 0.0747 | 0.021 |  |  |  |  |  |
| *G- Treponema* | 0.089 | 0.769 | 0.969 | 0.021 | 0.1611 | 0.044 | 0.005 |  |  |  |  |
| *G- Faecalibacterium* | 0.059 | 0.143 | 0.485 | 0.138 | 0.0478 |  | 0.004 |  |  |  |  |
| *G- Christensenellaceae_R-7_group* | 0.065 | 0.341 | 0.699 | 0.127 | 0.1125 |  | 0.013 |  |  |  |  |
| *G- Agathobacter* | 0.113 | 0.263 | 0.470 | 0.174 | 0.0566 |  | 0.013 |  |  |  |  |
| ***Taxa*** | **CON/CON 1** | **CON/PRO**  **2** | **PRO/CON 3** | **PRO/PRO**  **4** | **SEM^1^** | **Contrasts –*P* values** | | | | | |
|  |  |  |  |  |  | **1 vs 2** | **1 vs 3** | **1 vs 4** | **2 vs 3** | **2 vs 4** | **3 vs 4** |
| *G-Allorhizobium-Neorhizobium-Pararhizobium-Rhizobium* | 0.468 | 0.018 | 0.022 | 0.013 | 0.0530 |  | 0.014 | <0.001 |  |  |  |
| *G- Roseburia* | 0.020 | 0.077 | 0.223 | 0.090 | 0.0296 |  | 0.018 |  |  |  |  |
| *G- Fusicatenibacter* | 0.015 | 0.016 | 0.080 | 0.006 | 0.0099 |  | 0.018 |  |  |  |  |
| *G- Corynebacterium* | 0.292 | 0.067 | 0.042 | 0.039 | 0.0328 |  | 0.022 |  |  |  |  |
| *G- Blautia* | 0.616 | 0.445 | 1.823 | 0.932 | 0.2811 |  | 0.022 | 0.002 |  |  |  |
| *G- Megasphaera* | 0.121 | 0.185 | 0.555 | 0.069 | 0.0640 |  | 0.004 |  |  |  |  |
| *G- Prevotella* | 1.054 | 2.618 | 4.427 | 0.203 | 0.4863 |  | 0.004 |  |  |  |  |
| *G- Prevotellaceae_UCG-003* | 0.095 | 0.197 | 0.356 | 0.184 | 0.0505 |  | 0.031 |  |  |  |  |
| *G- Anaerovibrio* | 0.108 | 0.197 | 0.577 | 0.057 | 0.0640 |  | 0.006 |  |  |  |  |
| *G- Butyricicoccaceae_UCG-008* | 0.170 | 0.318 | 0.855 | 0.343 | 0.1079 |  | 0.004 |  |  |  |  |
| *G- Oscillospiraceae_NK4A214_group* | 0.025 | 0.188 | 0.249 | 0.030 | 0.0401 |  | 0.009 |  |  |  |  |
| *G- Oscillospiraceae_UCG-002* | 0.033 | 0.313 | 0.510 | 0.012 | 0.0796 | 0.044 | 0.004 |  |  |  | 0.030 |
| *G- Lachnospiraceae_AC2044_group* | 0.014 | 0.044 | 0.095 | 0.042 | 0.0132 |  | 0.026 |  |  |  |  |
| *G- Lachnospiraceae_ND3007_group* | 0.027 | 0.034 | 0.127 | 0.112 | 0.0189 |  | 0.050 |  |  |  |  |
| *G- Fournierella* | 0.011 | 0.037 | 0.200 | 0.043 | 0.0242 |  | 0.004 |  |  |  |  |
| *G- Rikenellaceae_dgA-11_gut_group* | 0.021 | 0.083 | 0.148 | 0.001 | 0.0171 |  | 0.037 |  |  |  |  |
| *G- Solobacterium* | 0.037 | 0.045 | 0.125 | 0.077 | 0.0190 |  | 0.023 |  |  |  |  |
| *G- Turicibacter* | 0.777 | 1.950 | 4.946 | 1.840 | 0.6849 |  | 0.009 |  |  |  |  |
| *G- Catenibacterium* | 0.155 | 0.149 | 1.126 | 0.160 | 0.1600 |  | 0.027 |  |  |  |  |
| *G- Catenisphaera* | 0.014 | 0.019 | 0.082 | 0.018 | 0.0091 |  | 0.026 |  |  |  |  |
| *G- Clostridium_sensu_stricto_6* | 0.015 | 0.050 | 0.114 | 0.214 | 0.0390 |  | 0.007 |  |  |  |  |
| *G- Rothia* | 2.169 | 0.715 | 0.396 | 0.332 | 0.1876 |  | 0.038 | 0.037 |  |  |  |
| *G- Phascolarctobacterium* | 0.177 | 0.272 | 0.835 | 0.075 | 0.0762 |  | 0.006 |  |  |  |  |
| *G- Parabacteroides* | 0.033 | 0.128 | 0.393 | 0.111 | 0.0418 |  | 0.007 |  |  |  |  |
| *G- Succinivibrio* | 0.055 | 0.162 | 0.309 | 0.021 | 0.0500 |  | 0.015 |  |  |  |  |
| *G- Subdoligranulum* | 0.090 | 0.180 | 0.445 | 0.340 | 0.0646 |  | 0.007 |  |  |  |  |
| *G- Collinsella* | 0.035 | 0.054 | 0.142 | 0.130 | 0.0319 |  | 0.007 |  |  |  |  |
| ***Taxa*** | **CON/CON 1** | **CON/PRO**  **2** | **PRO/CON 3** | **PRO/PRO**  **4** | **SEM^1^** | **Contrasts –*P* value** | | | | | |
|  |  |  |  |  |  | **1 vs 2** | **1 vs 3** | **1 vs 4** | **2 vs 3** | **2 vs 4** | **3 vs 4** |
| *G- Camplyobacter* | 0.127 | 0.132 | 0.604 | 0.540 | 0.1342 |  | 0.025 |  |  |  |  |
| *G- Alloprevotella* | 0.432 | 0.549 | 1.522 | 0.292 | 0.1707 |  | 0.050 |  |  |  | 0.029 |
| *G- Coprococcus* | 0.068 | 0.123 | 0.447 | 0.193 | 0.0569 |  | 0.007 |  |  |  |  |
| *G- Ruminococcus* | 0.024 | 0.195 | 0.270 | 0.085 | 0.0375 |  | 0.010 |  |  |  |  |
| *G- Sphaerochaeta* | 0.010 | 0.072 | 0.084 | 0.000 | 0.0138 |  | 0.022 |  |  |  |  |
| *G- Holdemanella* | 0.074 | 0.070 | 0.334 | 0.181 | 0.0477 |  | 0.007 |  |  |  |  |
| *G- Clostridium_sensu_stricto_1* | 5.096 | 6.649 | 4.275 | 12.229 | 1.6031 |  |  | 0.012 |  |  |  |
| *G- Prevotellaceae_NK3B31_group* | 0.203 | 1.127 | 1.472 | 0.020 | 0.2126 |  | 0.006 |  |  | 0.002 | 0.029 |
| *G- Family_XIII_AD3011_group* | 0.015 | 0.055 | 0.115 | 0.018 | 0.0174 |  | 0.041 |  |  |  |  |
| *G- Helcococcus* | 0.060 | 0.040 | 0.033 | 0.002 | 0.0077 |  |  | 0.009 |  |  |  |
| *G- Enhydrobacter* | 0.314 | 0.052 | 0.109 | 0.013 | 0.0487 |  |  | 0.037 |  |  |  |
| *G- Gemella* | 0.206 | 0.052 | 0.089 | 0.024 | 0.0162 |  |  | 0.037 |  |  |  |
| *G- Terrisporobacter* | 3.222 | 3.737 | 4.438 | 7.707 | 1.1116 |  |  | 0.037 |  |  |  |
| **Caecum d8 PW** | | | | | | | | | | | |
| *F- Oxalobacteraceae* | 0.195 | 0.089 | 1.911 | 0.841 | 0.2475 |  |  |  | 0.009 |  |  |
| *F- Paludibacteraceae* | 0.110 | 0.170 | 0.188 | 0.312 | 0.0439 |  | 0.017 |  |  |  |  |
| *F- Bacteroidales-p2534-18B5-gut-group* | 0.085 | 0.107 | 0.725 | 0.226 | 0.0965 |  | 0.027 |  |  |  |  |
| *G- Christensenellaceae_R-7_group* | 0.065 | 0.227 | 0.412 | 0.197 | 0.0564 |  | 0.034 |  |  |  |  |
| *G- Treponema* | 0.977 | 1.141 | 2.945 | 1.597 | 0.2675 |  | 0.037 |  |  |  |  |
| *G- Oscillospiraceae_NK4A214_group* | 0.095 | 0.113 | 0.238 | 0.098 | 0.0241 |  | 0.034 |  |  |  |  |
| ***Rectum d8 PW*** |  |  |  |  |  |  |  |  |  |  |  |
| *P- Campilobacterota* | 0.962 | 2.847 | 1.045 | 0.987 | 0.2594 | 0.006 |  |  | 0.003 | 0.003 |  |
| *P- WPS-2* | 0.197 | 0.063 | 0.202 | 0.037 | 0.0420 |  |  |  | 0.024 |  |  |
| *F- Campylobacteraceae* | 0.830 | 2.736 | 0.886 | 0.882 | 0.2529 |  |  |  | 0.030 | 0.035 |  |
| **Faeces d27 PW** | | | | | | | | | | | |
| *P- Firmicutes* | 54.268 | 47.845 | 53.786 | 55.891 | 1.2625 |  |  |  | 0.026 | 0.044 |  |
| *P- Actinobacteriota* | 0.576 | 0.410 | 0.327 | 0.556 | 0.0452 |  |  |  |  |  | 0.042 |
| *P- Verrucomicrobiota* | 0.362 | 0.497 | 0.479 | 0.112 | 0.0840 |  |  |  |  |  | 0.006 |
| ***Taxa*** | **CON/CON 1** | **CON/PRO**  **2** | **PRO/CON 3** | **PRO/PRO**  **4** | **SEM^1^** | **Contrasts –*P* values** | | | | | |
|  |  |  |  |  |  | **1 vs 2** | **1 vs 3** | **1 vs 4** | **2 vs 3** | **2 vs 4** | **3 vs 4** |
| *G- Lachnospiraceae_ND3007_group* | 0.261 | 0.149 | 0.122 | 0.023 | 0.0220 |  |  | <0.001 |  | 0.020 |  |
| *G- Prevotellaceae_UCG-001* | 0.365 | 0.646 | 1.126 | 0.937 | 0.2114 |  | 0.005 | 0.002 |  |  |  |
| ***Faeces d56 PW*** |  |  |  |  |  |  |  |  |  |  |  |
| *G- Alloprevotella* | 1.292 | 1.782 | 2.038 | 0.788 | 0.1451 |  |  |  |  |  | 0.025 |
| *F-Bacteroidales_RF16_group* | 0.018 | 0.043 | 0.927 | 0.031 | 0.1283 |  | <0.001 |  | <0.001 |  | <0.001 |
| **Faeces d118 PW** | | | | | | | | | | | |
| *F-Bacteroidales-p2534-18B5-gut -group* | 0.295 | 2.982 | 0.238 | 0.920 | 0.3666 | 0.009 |  |  | <0.001 |  |  |
| *F-Lactobacillicaceae* | 4.143 | 3.351 | 12.159 | 7.033 | 1.2104 |  | 0.033 |  | <0.001 |  |  |
| *G- Lactobacillus spp.* | 4.143 | 3.351 | 12.159 | 7.033 | 1.2104 |  | 0.005 |  | 0.007 |  |  |
| *G- Muribaculaceae_CAG-873* | 0.236 | 0.535 | 0.093 | 0.124 | 0.0542 |  |  |  | 0.007 |  |  |
| *G- Succinivibrio* | 1.026 | 1.154 | 0.257 | 0.674 | 0.2308 |  |  |  | 0.038 |  |  |
| *G- Catenisphaera* | 0.057 | 0.142 | 0.029 | 0.041 | 0.0180 |  |  |  | 0.007 |  |  |
| *G- Lachnospiraceae_NK3A20_group* | 0.062 | 0.097 | 0.027 | 0.084 | 0.016 |  |  |  | 0.005 |  |  |
| *G- Lachnospiraceae_NK4A136_group* | 1.302 | 1.340 | 0.403 | 0.387 | 0.2402 |  |  |  | 0.025 |  |  |
| *G- Asteroleplasma* | 0.166 | 0.300 | 0.124 | 0.088 | 0.0280 |  |  |  |  | 0.015 |  |
| *G- Roseburia* | 0.160 | 0.117 | 1.043 | 0.770 | 0.1390 |  |  |  | 0.021 |  |  |

SEM, standard error of the mean; G, genus; P, phylum; d, day; PW, post-weaning.

Treatments are as follows (maternal treatment/post-weaning treatment): CON/CON, CON/PRO, PRO/CON and PRO/PRO; where CON = control and PRO = probiotic.^1^Pooled standard error of the mean.

Statistical analysis of OTU abundance was performed using DeSeq2^26^ in R version 4.02^24^, where low abundance ASVs were manually filtered and a false discovery rate of < 0.05 was indicative of significant abundance difference between groups. For each taxon, differences between the median abundances of samples in each treatment group were assessed using the Wilcoxon Rank Sum test of the R package Metacoder^27^

**Supplementary Table S4. Composition of experimental diets (on an air-dry basis; kg/tonne unless otherwise stated).**

| **Item** | **Dry Sow** | **Lactating Sow** | **Starter/ link** | **Weaner** | **Finisher** |
| --- | --- | --- | --- | --- | --- |
| Barley | 753.02 | 269.81 | 62.86 | 257.58 | 384.67 |
| Wheat | 0 | 429.6 | 112 | 433.57 | 400 |
| Maize | 0 | 0 | 300 | 0 | 0 |
| Soybean meal | 89.62 | 196.65 | 255 | 187.92 | 183.01 |
| Soya hulls | 121.8 | 0 | 0 | 0 | 0 |
| Full fat soya | 0 | 0 | 70 | 50 | 0 |
| Lactoflo^1^ | 0 | 0 | 100 | 0 | 0 |
| Skim milk powder | 0 | 0 | 25 | 0 | 0 |
| Soya oil | 11 | 66 | 40 | 40 | 9.69 |
| Lysine HCl | 2.19 | 4.47 | 5.14 | 5.02 | 3.75 |
| DL-Methionine | 0.58 | 1.35 | 2.62 | 1.85 | 0.93 |
| L-Threonine | 0.6 | 2.45 | 2.55 | 2.09 | 1.7 |
| L-Tryptophan | 0 | 0.71 | 0.97 | 0.27 | 0.15 |
| L-Valine | 0 | 2.34 | 0.26 | 0 | 0 |
| Vitamin and mineral mix | 1.5^2^ | 1.5^2^ | 3^3^ | 3^3^ | 1^4^ |
| Salt feed grade | 4 | 5 | 3 | 3 | 3 |
| Mono di-calcium phosphate | 6.49 | 8.5 | 9.5 | 4.6 | 1 |
| Limestone flour | 9.08 | 11.5 | 8 | 11 | 11 |
| Phytase^5^ | 0.1 | 0.1 | 0.1 | 0.1 | 0.1 |
| **Analysed chemical composition** | | | | | |
| Dry matter | 875 | 898 | 891 | 897 | 876 |
| Crude protein | 129 | 164 | 190 | 193 | 171 |
| Fat | 36.6 | 102.8 | 65.1 | 72.1 | 43.5 |
| Crude fibre | 72 | 26 | 30 | 27 | 31 |
| Neutral detergent fibre | 162 | 82 | 88 | 84 | 103 |
| Ash | 40 | 48 | 48 | 45 | 43 |
| Lysine | 8.2 | 11.5 | 15.0 | 13.0 | 11.0 |
| Methionine | 2.7 | 3.8 | 5.8 | 4.6 | 3.5 |
| Methionine and cysteine | 5.4 | 7.0 | 9.1 | 7.9 | 6.7 |
| Threonine | 5.5 | 8.3 | 10.1 | 8.6 | 7.7 |
| Tryptophan | 1.7 | 2.8 | 3.4 | 2.6 | 2.3 |
| **Calculated chemical composition^6^** | | | | | |
| Standardised ileal digestible lysine | 6.60 | 10.67 | 14.00 | 11.49 | 9.97 |
| Calcium | 7.20 | 8.32 | 8.00 | 7.25 | 6.59 |
| Digestible phosphorus | 3.45 | 3.88 | 4.44 | 3.32 | 2.55 |
| Digestible energy (MJ/kg) | 13.2 | 15.2 | 15.0 | 14.5 | 13.8 |
| Net energy (MJ/kg) | 8.9 | 10.9 | 10.74 | 10.55 | 9.80 |

^1^Lactoflo 70 contains 70% lactose, 11.5% protein, 0.5% oil, 7.5% ash and 0.5% fibre (Volac, Cambridge, UK).

^2^Premix provided per kg of complete diet: Cu, 15 mg; Fe, 70 mg; Mn, 62 mg; Zn, 80 mg; I, 0.6 mg; Se, 0.2 mg; vitamin A, 1000 IU; vitamin D_3_, 1000 IU; vitamin E, 100 IU; vitamin K, 2 mg; vitamin B_12_, 15 μg; riboflavin, 5 mg; nicotinic acid, 12 mg; pantothenic acid, 10 mg; choline chloride, 500 mg; biotin, 200 mg; folic acid, 5 g; vitamin B_1_, 2 mg; vitamin B_6_, 3 mg.

^3^Premix provided per kg of complete diet: Cu, 155 mg; Fe, 90 mg; Mn, 47 mg; Zn, 120 mg; I, 0.6 mg; Se, 0.3 mg; vitamin A, 6000 IU; vitamin D_3_, 1000 IU; vitamin E, 100 IU; vitamin K, 4 mg; vitamin B_12_, 15 μg; riboflavin, 2 mg; nicotinic acid, 12 mg; pantothenic acid, 10 mg; choline chloride, 250 mg; vitamin B_1_, 2 mg; vitamin B_6_, 3 mg; Endox, 60 g.

^4^Premix provided per kg of complete diet: Cu, 15 mg; Fe, 24 mg; Mn, 31 mg; Zn, 80 mg; I, 0.3 mg; Se, 0.2 mg; vitamin A, 2000 IU; vitamin D_3_, 500 IU; vitamin E, 40 IU; vitamin K, 4 mg; vitamin B_12_, 15 μg; riboflavin, 2 mg; nicotinic acid, 12 mg; pantothenic acid, 10 mg; vitamin B_1_, 2 mg; vitamin B_6_, 3 mg.

^5^The diet contained 500 phytase units (FYT) per kg feed from RONOZYME HiPhos (DSM, Belfast, UK).

^6^Calculated from tabulated ingredient values (Sauvant et al., 2004)

**References**

Sauvant, D. *et al.* Tables of composition and nutritional value of feed materials : pigs, poultry, cattle, sheep, goats, rabbits, horses and fish. (2004).
